# Visual contrast from background features and dynamic illumination contributes to three-dimensional camouflage in cuttlefish

**DOI:** 10.1242/jeb.249713

**Published:** 2025-08-15

**Authors:** Christian Drerup, Martin J. How, James E. Herbert-Read

**Affiliations:** ^1^ Department of Zoology, University of Cambridge, Cambridge CB2 3EJ, UK; ^2^ Department of Biosciences, Durham University, Durham DH1 3LE, UK; ^3^ School of Biological Sciences, University of Bristol, Bristol BS8 1TQ, UK

**Keywords:** Caustics, Cephalopod, Dynamic lighting, Sensory ecology, *Sepia officinalis*

## Abstract

Many animals adopt camouflage strategies that involve matching their appearance to colour and texture-based features in their environment. However, these features may be difficult to estimate in habitats that are prone to dynamic lighting, which might alter the features' appearance, or disrupt the capacity of visual systems to resolve those features. In this study, we tested whether a common form of shallow underwater dynamic lighting termed ‘caustics’, consisting of moving light bands travelling along the substrate, affect the expression of skin papillae in cuttlefish (*Sepia officinalis*). To do so, we exposed cuttlefish individuals to rock stimuli varying in their surface texture and colouration in both caustic and non-caustic lighting and scored their papillae expression. We established a positive correlation between the degree of papillae expression and the maximum contrast cues in the visual scene, such as those derived from object surface texture or colouration, with stronger contrast cues resulting in a more pronounced papillae expression. In addition, we found that cuttlefish also expressed their papillae when exposed to caustics, and this response was adopted irrespective of the presence or absence of an object in their visual field, highlighting that increased visual contrast levels deriving from exposure to dynamic lighting alone can elicit papillae expression in cuttlefish. We discuss whether these camouflage responses might be adaptive, reducing their likelihood of being detected by predators, or alternatively could represent a constraint on visual processing.

## Teaser

**Summary:** Cuttlefish express papillae based on contrast cues in their visual scene, but those contrast cues can derive from object-based features as well as from exposure to dynamic lighting.

## INTRODUCTION

Many animals have evolved camouflage strategies to avoid predation (Merilaita et al., 2017), for example through background matching (Merilaita and Stevens, 2011; Michalis et al., 2017) or displaying contrasting colour patterns to break up their body outline (‘disruptive colouration’) (Stevens et al., 2006, 2009). In addition to these camouflage strategies that commonly rely on specific body patterns, many animals have further evolved three-dimensional (3D) body protrusions for increased concealment (Yu et al., 2024). Several benthic fish species, for example, possess cutaneous appendages to match the 3D texture of objects, such as rocks, plants or algae, in their environment (Allen et al., 2015; Cott, 1940; Randall and Randall, 1960). Moreover, 3D body protrusions can also improve concealment of the body outline by decreasing edge intensity (Rohr et al., 2021).

One animal group capable of adjusting their 3D body protrusions in response to their environment is the cephalopods, with cuttlefish and octopus species being able to rapidly change the 3D texture of their skin through the expression of subcutaneous muscles (Allen et al., 2009, 2014). These so-called ‘skin papillae’ are activated by a neurally controlled muscular hydrostat system (Gonzalez-Bellido et al., 2018) that allows the size and shape of individual papillae to be varied (Allen et al., 2014). By expressing or retracting their papillae, these cephalopods can change their skin texture from smooth to three-dimensionally textured within seconds, allowing them to match the topography of various objects in their natural environments (Panetta et al., 2017).

Although cuttlefish change their papillae expression through visual rather than tactile inspection of their environment (Allen et al., 2009), the exact visual features eliciting the expression of skin papillae remain unknown. Some studies suggest that cuttlefish express their skin papillae in response to 3D features in their visual scene, such as rocks with algae filaments, thereby masquerading as these objects (Panetta et al., 2017). However, cuttlefish showed no differences in their papillae expression when exposed to natural substrate types offering 3D cues compared with 2D photographs of these substrate types which lack any 3D information (Allen et al., 2009). Therefore, 3D texture per se does not appear to be the sole driving visual feature eliciting papillae erection in cuttlefish. Despite the disparity in 3D cues between the natural backgrounds and the 2D photographs used by Allen et al. (2009), contrast cues based on chromatic and intensity differences within these substrates likely remained similar across stimuli, suggesting the possibility that cuttlefish express their papillae in response to patterns of visual contrast in the scene. Although contrast cues have been identified as some of the major visual cues used by cuttlefish to inform their body colouration (Barbosa et al., 2008; Chiao and Hanlon, 2001; Chiao et al., 2007; Drerup et al., 2024a), as well as in choosing whether to visually resemble 3D objects or the benthic background (Buresch et al., 2011), previous studies have not determined the role of visual contrast on cuttlefish papillae expression. Given that contrast might not only derive from chromatic and intensity differences across an object's surface colouration, but also from the 3D texture of the surface (where direct lighting produces brightly illuminated areas which stand in contrast to shaded regions on that object), this may explain previous observations of cuttlefish erecting their papillae in response to 3D textured objects (e.g. Hanlon and Messenger, 1988; Panetta et al., 2017).

**Fig. 1.**
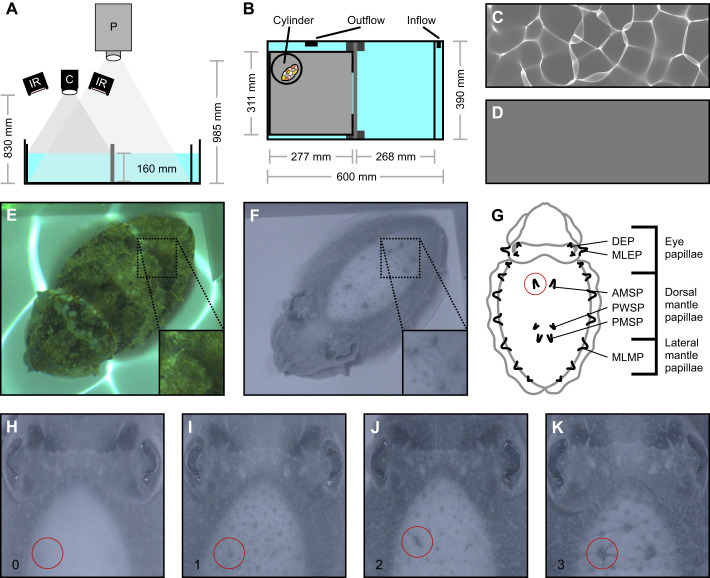
**Experimental setup.** (A,B) Schematic diagrams of the experimental arena. (A) Frontal view, with projector (P), camera (C) and infrared light (IR). (B) Top view. (C,D) Different lighting conditions used in this study, with (C) moving caustic pattern and (D) static uniform grey image. (E,F) Example images of a cuttlefish in the experimental arena taken through (E) a regular camera and (F) our modified IR camera, highlighting how IR photography removes the caustic projection from the images as well as facilitates easier identification of the cuttlefish skin papillae expression. (G) Papilla types scored in this study: dorsal eye papillae (DEP); major lateral eye papillae (MLEP); anterior mantle spot papillae (AMSP); posterior white square papillae (PWSP); posterior mantle spot papillae (PMSP); and major lateral mantle papillae (MLMP) (modified from Allen et al., 2009). (H–K) Representative examples of the scored expression levels of the left anterior mantle spot papillae (AMSP; red circles), consisting of (H) 0, no expression; (I) 1, weak expression; (J) 2, intermediate expression; and (K) 3, strong expression.

If cuttlefish alter their skin papillae expression in response to visual contrast cues, how is this camouflage behaviour then affected by environments prone to rapid changes in their visual contrast levels? Many cuttlefish species, such as the common cuttlefish, *Sepia officinalis*, inhabit shallow marine environments (∼2–10 m; Bloor et al., 2013a; Guerra, 2006), especially between spring and summer, when they migrate to shallow coastal areas (Bloor et al., 2013b; Drerup and Cooke, 2021). In these shallow habitats, light passing through the water's moving surface can be refracted in ways that create dynamically moving light patterns on the substrate, known as ‘caustics’ (Lock and Andrews, 1992; McFarland and Loew, 1983; see Fig. 1C as well as Movie 1 for visual reference), which can result in strong local fluctuations in the intensity of light (Drerup et al., 2024a; Venables et al., 2022). These intensity fluxes can spatially and temporarily alter the visual contrast levels in the scene, resulting in the adoption of disruptive body patterns in cuttlefish exposed to caustic lighting (Drerup et al., 2024a). Therefore, investigating whether dynamic lighting in the form of caustics also affects the 3D camouflage and papillae expression of cuttlefish might shed light on the mechanisms and visual features that cuttlefish use to inform their papillae erection.

Contrasting elements in natural environments, in the form of both true background features as well as dynamic lighting, will likely vary in their size, creating differences in the spatial frequencies or ‘granularity’ of the visual scene. Spatial properties are an important aspect for how animal body patterns are perceived by other animals and the effectiveness of camouflage strategies (Stoddard and Osorio, 2019). In cuttlefish, the spatial size of background features plays a key role in their body pattern expression (summarised in Chiao et al., 2015), with not only the size of discrete objects (Barbosa et al., 2008; Chiao et al., 2007; Chiao and Hanlon, 2001) but also the global spatial scale of the background texture affecting the body pattern expression in cuttlefish (Chiao et al., 2009). Moreover, cuttlefish use the spatial orientation of visual features to adapt their visual appearance to their habitat, for example by aligning their arm posture in relation to spatial patterns in their environment (Barbosa et al., 2011). When assessing the impacts of contrast from static object features and dynamic lighting on cuttlefish papillae expression, it is therefore important to also measure the impacts of these features on the spatial frequency in the visual scene to determine the likely drivers behind changes in papillae expression.

In this study, therefore, we asked whether the common cuttlefish, *S. officinalis*, erects its skin papillae in response to patterns of contrast and granularity in its visual scene, and whether the presence of dynamic lighting in the form of caustics affects the papillae expression of this species. We predicted that cuttlefish papillae expression could be affected in one of two ways. First, caustics might impair the visual or cognitive capacities of cuttlefish to correctly extract existing contrast cues of objects, thereby potentially reducing both the cuttlefish papillae expression in general, as well as their ability to match the textural appearance of their habitat. Alternatively, if cuttlefish erect their papillae based on visual contrast cues or spatial information in their vicinity, caustics might raise existing contrast or granularity levels in the scene, potentially promoting a stronger expression of skin papillae. In particular, caustics might increase the contrast between chromatic or achromatic elements across an object's surface, or alternatively raise the contrast deriving from different lighting conditions (direct illumination versus shaded areas) for three-dimensionally textured objects. We tested these predictions by establishing the degree of papillae expression in response to rock stimuli varying in texture (smooth versus textured) and colouration (grey versus multicoloured) under both caustic and non-caustic (‘static grey’) illumination, with both the texture and colouration of the rock, as well as the presence of caustic lighting, affecting the visual contrast levels.

## MATERIALS AND METHODS

### Animal husbandry

Common cuttlefish, *Sepia officinalis* Linnaeus 1758 (*n*=20, mantle length: 66±11 mm, mean±s.d.), were reared from eggs collected from the British South Coast at the laboratory of the Marine Biological Association (Plymouth, UK). Cuttlefish were kept in individual compartments (390×640 mm, 310 mm deep, water level at 250 mm) connected to a flow-through system supplied by natural seawater. All holding tanks were equipped with mesh lids and aerators, exposed to ambient light following the natural light regime of the experimental period (October), and contained rocks and artificial plants for enrichment. Cuttlefish were fed three times a day with live river shrimp (*Palaemon varians*) as well as once per week with live shore crabs (*Carcinus maenas*). All individuals were examined for signs of stress (based on locomotor, postural and chromatic displays), illness (e.g. injuries, changes in skin and eye appearance, unusual activity levels or ventilation rate) or inadequate nutrition (e.g. protruding eyes, poor body condition, floating) on a daily basis following established protocols (Andrews et al., 2013; Fiorito et al., 2015); however, none of the cuttlefish exhibited any of these signs throughout the experimental period.

### Experimental setup

The experimental arena (internal dimensions 600×390 mm, 355 mm deep, water level at 160 mm) was divided into a holding side (303×390 mm) and a test side (287×390 mm) (Fig. 1A,B). The divider consisted of an opaque, perforated Perspex sheet which could be raised, allowing cuttlefish to move from one side to another. On the holding side, we partitioned a 35 mm wide area using an opaque, perforated Perspex sheet to accommodate the inflow of natural seawater from the same flow-through system that supplied the cuttlefish holding tanks. Resting against the Perspex sheet was another identically sized, opaque and perforated Perspex sheet that, by slowly moving it sideways, could be used to reduce the size of the holding side to carefully coax cuttlefish over to the test side. On the test side, we partitioned a 277×311 mm wide area using opaque Perspex sheets with a 225×150 mm wide opening on the side facing the vertical divider, which restricted the cuttlefish to a defined area holding the rock stimuli (Fig. 1B). This subdivision of the test side further created two smaller sections, one of which held the outflow (Fig. 1B). All Perspex sheets used in this setup were perforated to facilitate the water flow throughout the arena. A black fabric cubicle surrounded the entire setup to maintain consistent ambient light conditions and minimise external disturbances.

We suspended a projector (CP-WX3541WN, Hitachi Ltd, Tokyo, Japan) 985 mm above the arena (Fig. 1A), which was used to cast a computer-generated animation of a moving caustic pattern (Fig. 1C; Movie 1) or a static uniform grey image (Fig. 1D) over the whole arena. The caustic animation was rendered using Caustics Generator Pro software (Dual Heights; www.dualheights.se/caustics/) and consisted of 200 unique frames that were looped at a playback speed of 30 frames s^−1^, resulting in a full loop every 6.67 s (for software settings, see Drerup et al., 2023, 2024b). For the static uniform grey image, a unit8 value of 87 was chosen to match the same average light intensity as the caustic animation (Fig. S1A,B).

We mounted a modified DSLR camera (D7100, Nikon Corporation, Tokyo, Japan) 830 mm above the arena (Fig. 1A). This camera had its internal hot mirror filter exchanged for a custom-made long-pass infrared (IR) filter provided by Lifepixel (https://www.lifepixel.com/), thereby only allowing IR light >720 nm to be captured by the camera. Two IR lights were mounted above the arena at the same height as the camera, casting IR light into the test side of the arena (Fig. 1A). As with most marine organisms, cuttlefish are insensitive to IR light (Brown and Brown, 1958), and previous studies have shown that cuttlefish do not alter their behaviour under IR light (e.g. Allen et al., 2010; Brauckhoff et al., 2020; Wu et al., 2020). The IR camera was connected to a laptop (G3, Dell Technologies Inc., Round Rock, TX, USA) and controlled using OBS Studio (https://obsproject.com/), allowing us to remotely take IR images of the cuttlefish within the test side of the arena (Fig. 1F). Using an IR camera instead of a regular camera yielded two benefits. First, images taken in IR did not include the caustic pattern projected into the arena (Fig. 1E,F), as the projector's image is restricted to the visible light spectrum. Second, IR images of cuttlefish do not contain any indication of their colour patterns, likely because the chromatophore pigments are transparent to wavelengths >720 nm (Fig. 1E,F). Taking IR images thus facilitated the scoring of the skin papillae expression blind to the presence or absence of body patterns or lighting condition information.

### Rock stimuli

**Fig. 2.**
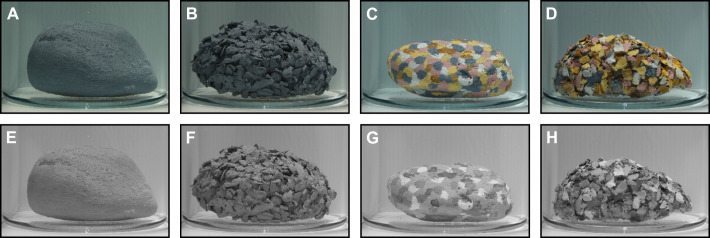
**Representative images of the four tested rock stimuli.** (A) Grey–smooth. (B) Grey–textured. (C) Coloured–smooth. (D) Coloured–textured. (E–H) Same images as in A–D but visually modelled to cuttlefish vision. When taking the images presented in A–D, both the arena as well as the glass cylinder were filled with saltwater, highlighting that placing the rocks in the cylinder did not result in any obvious type of visual distortion that might alter their appearance to the cuttlefish.

We used rock stimuli varying in their surface texture (smooth versus textured) and colouration (grey versus multicoloured) (Fig. 2A–D) to elicit camouflage behaviour in cuttlefish. To create these stimuli, we collected 12 rocks that were approximately 80 mm long, roughly similar in their shapes and volume, and with a smooth surface. To half of these stones, we attached small-scale fragments (<10 mm) of oyster shells using aquarium-safe silicone, thereby changing the rock topography from smooth to textured. We painted three smooth and three textured rocks with a uniformly grey oil-based paint (Fig. 2A,B). The remaining six rocks (three smooth and three textured) were painted with oil-based paint of different shades (Fig. S1C) mirroring common colour shades found in shallow marine habitats (Fig. 2C,D). As cuttlefish appear to be colourblind (Marshall and Messenger, 1996; Mäthger et al., 2006; but see Stubbs and Stubbs, 2016), they likely perceive these different colour shades (and any naturally occurring chromatic differences in their wild habitat) solely as differences in intensity, creating achromatic contrast in their visual scene (Fig. 2E–H), which appears to be an important visual feature to inform their body pattern expression for camouflage (Chiao et al., 2015; Drerup et al., 2024a). For the coloured–textured rocks, we painted each shell fragment individually so that neighbouring fragments did not share the same colouration (Fig. 2D), whereas for the coloured–smooth rocks, we painted small patches of paint (<10 mm) across each rock, mimicking the pattern of the coloured–textured rocks (Fig. 2C). These steps yielded four different types of rock stimuli with three replicates each: (i) grey–smooth (Fig. 2A); (ii) grey–textured (Fig. 2B); (iii) coloured–smooth (Fig. 2C); and (iv) coloured–textured (Fig. 2D). To prevent cuttlefish from resting on top of the rocks or gaining tactile information about the rock surface topography (but see Allen et al., 2009), all rocks were presented in a glass cylinder (120 mm diameter, 180 mm height) filled with seawater, which further served as (v) a control stimulus by presenting it to the cuttlefish without a rock inside. Placing the painted rock stimuli inside the glass cylinder further ensured that cuttlefish were not exposed to any potential solvents that might harm them or alter their behaviour. Moreover, because the glass cylinder was both filled and immersed in water (thereby minimising the refraction of light passing through the glass), the visual appearances of the rock stimuli were relatively undistorted from a cuttlefish's perspective (Fig. 2A–D).

### Experimental protocol

A trial consisted of exposing a cuttlefish to (i–iv) all four rock stimuli as well as (v) the empty glass cylinder control. All cuttlefish (*n*=20) were exposed to the five stimuli in two trials, once in static grey light and once in the caustic condition, resulting in 40 trials in total. To ensure that the order of the five stimuli within a trial did not affect the cuttlefish camouflage response, we created 20 unique orders of the five stimuli per lighting condition in a randomised block design. Therefore, each cuttlefish received a unique order of the presented stimuli in both in the static grey treatment and the caustic treatment. Half of the cuttlefish (*n*=10) first received the static grey treatments, followed by the caustic treatments, whereas the other half of the tested individuals received these treatments in the opposite order.

For each trial, either the empty glass cylinder or the glass cylinder containing a rock stimulus was placed in the far-right corner of the test enclosure (from the perspective of the cuttlefish entering). Subsequently, a cuttlefish was introduced into the holding side of the arena, and the corresponding lighting condition (caustic animation or static uniform grey image) was started remotely. Each cuttlefish was given 10 min to acclimatise in the holding side before the arena divider was lifted and the cuttlefish was carefully coaxed into the test side using the Perspex sheet already present in the holding side. By slowly moving this Perspex sheet sideways, we continuously reduced the size of the holding side, thereby encouraging cuttlefish to move over to the test side. This approach, which was identically executed for each treatment, ensured that we did not introduce any new objects into the arena or directly interfere with a cuttlefish, thereby minimising the risk of eliciting any forms of unnatural behaviour in the cuttlefish that might affect their papillae expression. After lowering the divider, the cuttlefish was exposed to a stimulus for 5 min. At the end of this period, the divider was slowly lifted again, and the cuttlefish was coaxed back to the holding side, followed by lowering the divider again. Subsequently, the stimulus within the test side was exchanged for the following stimulus, after which the cuttlefish was given another 5 min in the holding side before lifting the divider again and exposing the cuttlefish to the next stimulus. This procedure was repeated throughout the whole trial, resulting in a total trial length of less than 1 h (10 min acclimatisation, 5×5 min exposure to stimuli, 4×5 min resting in holding side between stimuli exposures, plus 4× roughly 1 min to exchange stimuli) per cuttlefish.

Pilot observations conducted in advance of this experiment revealed that once a cuttlefish rested next to a rock stimulus, papillae expression occurred rapidly (<5 s, see also Panetta et al., 2017) and was stable over time, regardless of the lighting condition (Fig. S2). In line with our previous study establishing that body pattern expression in stationary cuttlefish exposed to caustic lighting becomes stable after 25–30 s (Drerup et al., 2024a), we decided to also score the papillae expression after this time period. Within each 5-min-long exposure period of a cuttlefish to a rock stimulus, we therefore remotely monitored the cuttlefish activity for stationary periods using the video stream of the IR camera displayed on the connected laptop. The first time a cuttlefish was stationary for 30 s within one body length of the glass cylinder, an image was taken for subsequent analysis of the cuttlefish's papillae expression. This approach should have resulted in 200 images (20 cuttlefish×5 stimuli×2 lighting conditions). However, in approximately 29% of the trials the cuttlefish did either not rest long enough or not within one body length of the glass cylinder, so no images could be acquired, resulting in a total of 141 images obtained for subsequently analysis.

### Papillae scoring

IR images were prepared for scoring by cropping each image so that the rock stimulus was removed. This process, in combination with taking images of the cuttlefish in IR, which therefore did not contain any indication about the lighting condition (presence or absence of caustics) or cuttlefish pattern, allowed us to score the cuttlefish skin papillae expression unbiased by the treatment. We established the papillae expression by scoring the following papilla types (for a detailed description, see Allen et al., 2009; Panetta et al., 2017): dorsal eye papillae (DEP); major lateral eye papillae (MLEP); anterior mantle spot papillae (AMSP); posterior white square papillae (PWSP); posterior mantle spot papillae (PMSP); and major lateral mantle papillae (MLMP) (Fig. 1G). Following Panetta et al. (2017), we scored each papilla type using the following scale: 0, no expression; 1, weak expression, approximately 1/3 extended; 2, intermediate expression, approximately 2/3 extended; and 3, strong expression, fully extended. As the expression of all six papilla types was positively correlated (see Results), we established for each cuttlefish a ‘total papillae expression score’ by calculating the sum of all six individual papillae expression scores. This scoring system could therefore result in total papillae expression scores between 0 (no papillae expressed) and 18 (all six papilla types fully extended). Considering that the view on the papillae on the cuttlefish body side facing the rock stimuli were often obscured for cuttlefish resting very close to the glass cylinder, only the papillae on the side of the body non-adjacent to the rock stimuli were scored. All 141 images were scored by the same observer. In addition, a subset of 30 images (∼21% of full dataset) was further scored by a second, independent observer who was also completely blind to the treatments. Interobserver reliability was assessed by establishing the level of agreement between both observers using a weighted Cohen's kappa test (McHugh, 2012). This analysis was performed in R v. 4.3.2 (https://www.r-project.org/) using the irr package (https://CRAN.R-project.org/package=irr) and resulted in a Cohen's kappa value of 0.91, which is considered an almost perfect agreement between observers (McHugh, 2012). Therefore, we deemed the papillae expression scores of the first observer reliable and used those for subsequent analysis.

### Monochromatic images

To quantify the visual input used by cuttlefish to elicit papillae expression, we recreated our experimental setup using a small glass aquarium (internal dimensions 400×200 mm, 200 mm deep), filled to 160 mm with artificial seawater (salinity 35 ppm). We obscured both long and one short side of the aquarium with opaque Perspex sheets and mounted the same projector (CP-WX3541WN) above the aquarium at the same height (985 mm) as in the experiment. We then placed the water-filled glass cylinder, either empty or holding one of the stimulus rocks, in the left corner opposite the unobscured aquarium wall. Using an unmodified DSLR camera (EOS 5D Mark IV, Canon Inc., Tokyo, Japan) in manual mode at constant settings (6720×4480 pixels, f/22, 1/10 s, ISO-400), we took five RAW images of each rock replicate (*n*=12) as well as the empty cylinder, each exposed to five different still frames of caustic projection, and one RAW image of each stimulus replicate in non-caustic (static-grey) lighting, resulting in a total of 78 images. Considering physiological and behavioural data indicate that cuttlefish are colourblind (Marshall and Messenger, 1996; Mäthger et al., 2006), all images were converted into monochromatic images, approximating how a cuttlefish would perceive each rock (Fig. 2E–H). As the maximum absorption of the cuttlefish monochromatic visual pigment lies at 492 nm (Brown and Brown, 1958), and considering that the spectral sensitivity of the camera used in our setup is approximately equally distributed between the G- and B-channels at this wavelength, we converted all images to cuttlefish vision using a bespoke MATLAB code that involved omitting the R-channel of each image and taking an average of each pixel from the G- and B-channels.

### Contrast analysis

To establish whether cuttlefish erect their papillae in response to contrast cues in the visual scene, we established proxies for the average and maximum contrast cues of each stimulus rock in either caustic or non-caustic lighting conditions. Using the monochromatic images, we rendered 10 horizontal and 10 vertical transect lines within the outline of each rock image, all equally spaced and 1 pixel wide. In our experiment, caustics were projected only onto the bottom of the arena, so for the images containing the cylinder alone we highlighted a rectangular bounding box similar in size to the rocks onto the bottom of the arena and rendered 20 transects within this box as described above. Using the width of the glass cylinder in each image as a size reference, we then divided each transect into 5 mm long segments and calculated the mean pixel intensity of each transect segment. We chose a segment length of 5 mm as this represented the lower size range of the shell fragments, and calculated the Michelson contrast (MC) between neighbouring transect segments using the equation:
(1)

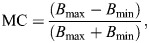
where *B*_max_ and *B*_min_ refer to the maximum and minimum mean pixel values measured, respectively. We then established an average contrast proxy for each type of rock stimulus in either non-caustic or caustic conditions by calculating the mean of all obtained MCs for neighbouring transect segments across all transects of all replicates, either in caustic or non-caustic conditions. Similarly, we also established a maximum contrast proxy for each type of rock stimulus in either non-caustic or caustic conditions by determining for each pair of neighbouring transect segment the highest MC across all replicate measurements. We opted to use the MC as our contrast measurement as it has been shown to be a major visual feature for cuttlefish to inform their camouflage strategies (e.g. Barbosa et al., 2008; Buresch et al., 2011; Chiao and Hanlon, 2001; Drerup et al., 2024a; Kelman et al., 2007; Mäthger et al., 2006; Zylinski et al., 2009).

### Granularity analysis

Our experimental approach was designed to minimise the effect of spatial cues impacting the cuttlefish papillae expression, with all shell fragments and coloured patches being roughly the same size. As small differences in the size of the shell fragments or coloured patches might still result in differences in the spatial frequency of the rock stimuli, we conducted a granularity analysis of each stimulus rock in either caustic or non-caustic lighting conditions to establish whether cuttlefish erect their papillae in response to the spatial information of their visual stimuli. Granularity analysis is a proxy for how animals process spatial information and is based on applying 2D fast Fourier bandpass filtering to an image (Godfrey et al., 1987). Here, each bandpass filter acts like a sieve, thereby allowing different spatial scales (so-called granularity bands) to be separated and individually analysed (Stoddard and Stevens, 2010). For each granularity band, the pattern ‘energy’ can then be established as the standard deviation of the filtered pixel values, with the energy across all granularity bands producing a ‘granularity spectrum’ (Chiao et al., 2009; Stoddard and Stevens, 2010). Granularity analysis is a common investigative feature for establishing spatial pattern properties (Stoddard and Osorio, 2019) and has previously been used to analyse animal body patterns (e.g. cuttlefish: Barbosa et al., 2008; fish: John et al., 2024; moths: Mark et al., 2022; lizards: Wuthrich et al., 2022) or egg shell patterns (Quach et al., 2021; Stoddard and Stevens, 2010; Šulc et al., 2019).

By importing our 78 monochromatic images into the MICA toolbox (version 2.2.2; Troscianko and Stevens, 2015), a plugin of the image processing software ImageJ (Schneider et al., 2012), and creating a region of interest (ROI) for the outline of the rock in each image, we conducted a granularity analysis for each stimulus rock using MICA's inbuilt ‘Pattern Colour & Luminance Measurements’ function (Troscianko and Stevens, 2015). Using the fast Fourier transformation (FFT) bandpass method, we analysed 25 filter sizes (‘granularity bands’) ranging from 1 to 4096 pixels with stepwise increments of multiplies of √2 (van den Berg, 2018). From the resulting granularity spectra (Fig. S3), we obtained for each image the maximum frequency (‘maxFreq’; the spatial frequency with the highest energy, reflecting the most dominant spatial scale), the maximum energy (‘maxPower’; the energy within the maximum spatial frequency), as well as the total energy (‘sumPower’; sum of pattern energy across all filter sizes), which reflects the overall amplitude of the granularity spectrum and therefore provides a measure of the general spatial contrast of our stimulus rocks, with higher values indicating more spatially rich stimuli (Chiao et al., 2009; Stoddard and Stevens, 2010; van den Berg, 2018).

### Statistical analysis

All statistical analyses were performed in R v. 4.3.2. Model assumptions of all linear (mixed-effects) models were checked using the performance package (Lüdecke et al., 2021) and DHARMa package (https://cran.r-project.org/package=DHARMa). Statistical significance of fixed effects within a model were determined using the ‘drop1’ function from the lme4 package (Bates et al., 2015). All data visualisations were rendered using the ggplot2 package (Wickham, 2016) and ggrain package (Allen et al., 2021; https://cran.r-project.org/package=ggrain).

We first determined whether the expression of the six scored papilla types was correlated, allowing us to calculate an overall papillae expression score for subsequent analysis, or whether the six papilla types were expressed independently, thereby requiring an individual analysis for each papillae type. To do so, we established the Spearman's rank-based correlation coefficients between the six papilla types using the rstatix package (https://cran.r-project.org/package=rstatix), with all *P*-values being corrected for multiple testing using the Holm method. Owing to significantly positive correlations between all six papilla types (see Results), we continued the analysis using an overall ‘papillae expression score’, consisting of the sum of all six individuals’ papillae scores.

To investigate whether cuttlefish papillae expression was affected by either the different rock stimuli or the presence or absence of dynamic lighting in the form of caustics, we tested whether the total papillae expression score could be modelled as a function of stimulus rock type and lighting condition using a linear mixed model (LMM). We set the total papillae expression score as a continuous response variable, the rock stimulus and caustic level as a categorical fixed effects, and cuttlefish ID as a random effect owing to re-testing of the same individuals across multiple trials. We added an interaction term between the fixed effects (rock stimulus and caustic level) to determine whether the total papillae expression depended on the combination of exposure to a rock stimulus and the prevailing lighting condition. We used the ‘emmeans’ function from the emmeans package (https://CRAN.R-project.org/package=emmeans) to compute pairwise differences in the papillae expression between the 10 different treatments (five rock stimuli in two lighting conditions), with statistically similar treatments being assigned into groups using the ‘cld’ function from the multcomp package (Hothorn et al., 2008).

We investigated which contrast and granularity measurements taken from our treatments elicited papillae expression. To do so, we first established whether the average and maximum MC varies between rock stimuli, and whether exposure to caustics alters these contrast levels. To do so, we designed (i,ii) two linear models (LMs) with either (i) the average MC or (ii) maximum MC as a continuous response variable [in both instances Box–Cox transformed using the bestNormalize package (https://cran.r-project.org/package=bestNormalize) to meet normality assumptions], and the rock stimuli and caustic level as categorical fixed effects. Likewise, we tested whether the stimulus rocks varied in their maximum energy, maximum frequency or total energy, and whether exposure to caustics further affected these granularity measurements of the stimulus rocks. To do so, we designed (iii–v) three LMs with either (iii) the maximum energy, (iv) the maximum frequency or (v) the total energy measurements obtained from the granularity analysis as continuous response variables, and the rock stimuli and caustic level as categorical fixed effects. For all five models (i–v), we established groups of fixed effects with statistically similar contrast or granularity measurements using the ‘emmeans’ function from the emmeans package (https://CRAN.R-project.org/package=emmeans) and the ‘cld’ function from the multcomp package (Hothorn et al., 2008).

We then established which contrast or granularity measurement was the best predictor of papillae expression in cuttlefish. In other words, we asked which contrast or granularity measurement provided the most explanatory power to predict papillae expression. To do so, we ran (vi–x) five LMMs with the total papillae expression score as the response variable and cuttlefish ID as a random effect. Here, every model only contained a single contrast or granularity measurement as their fixed effect (vi: average contrast; vii: maximum contrast; viii: maximum energy; ix: maximum frequency; x: total energy). In addition, we further ran (xi) a null model with the same structure as models vi–x but not containing any fixed effects. We then performed an Akaike information criterion (AIC) model selection method using the AICcmodavg package (https://cran.r-project.org/package=AICcmodavg) to establish which of our contrast and granularity measurements best explained papillae expression in cuttlefish, with differences in the AICc of greater than 2 indicating strong support of one model over another (Burnham and Anderson, 2002).

We then tested whether cuttlefish papillae expression is best predicted by either one of the visual features we manipulated across all treatments (object presence, object surface texture, object surface colouration and caustic exposure), or the best supported (AICc<2) contrast or granularity measurements obtained from our model selection process above (maximum MC; see Results). To do so, we used model averaging, which is a common statistical approach in ecology to account for model selection uncertainty by combining estimates from multiple models rather than relying on a single ‘best’ model (Dormann et al., 2018). Instead of choosing a single model to describe which visual features or information cuttlefish use to erect their papillae, our model averaging process incorporated multiple candidate models based on a predefined global model and weighted each model variable according to its relative support of the data, thereby providing robust variable estimates to establish which visual cues best predict papillae expression in cuttlefish. In particular, we first designed a global linear mixed model with the papillae expression score as a continuous response variable, cuttlefish identity as a random effect due to re-testing of the same individuals, and the following variables as categorical, ordinal or continuous fixed effects: object presence (categorical: rock absent versus rock present), surface texture (ordinal: no surface<smooth surface<textured surface), surface colouration (ordinal: no colouration<single-coloured<multi-coloured), caustic exposure (categorical: no caustics versus caustics) and maximum contrast (continuous) [in R nomenclature: total papillae expression score∼object_presence+texture+colouration+caustic_exposure+maximum_contrast+(1|cuttlefishID)]. Using the arm package (https://cran.r-project.org/package=arm), we standardized the global model to facilitate interpretation of the subsequent analysis. We then assessed the effect of each explanatory variable (object presence, surface texture, colouration, caustic exposure, maximum contrast) by establishing model averaged coefficient estimates and 95% confidence intervals (CIs) using the MuMIn package (https://cran.r-project.org/package=MuMIn). We assessed candidate models which included all possible combinations of the explanatory variables using corrected AIC (AICc), and subsequently formed a selection of top models with a cumulative weight of 95% to perform the final model averaging. Additionally, we established the relative importance (RI) of each explanatory variable using the ‘sw’ function from the MuMIn package, with RI values ranging from 0 (parameter appears in the least supported models) to 1 (parameter appears in the best supported models) (Burnham and Anderson, 2002).

### Ethical note

The experiment outlined in this study adhered to the ASAB/ASB guidelines for use of animals in behavioural research, was conducted in accordance with UK legislation, and was approved by the University of Cambridge Animal Welfare and Ethical Review Body under UBS reference number NR2021/21.

## RESULTS

**Fig. 3.**
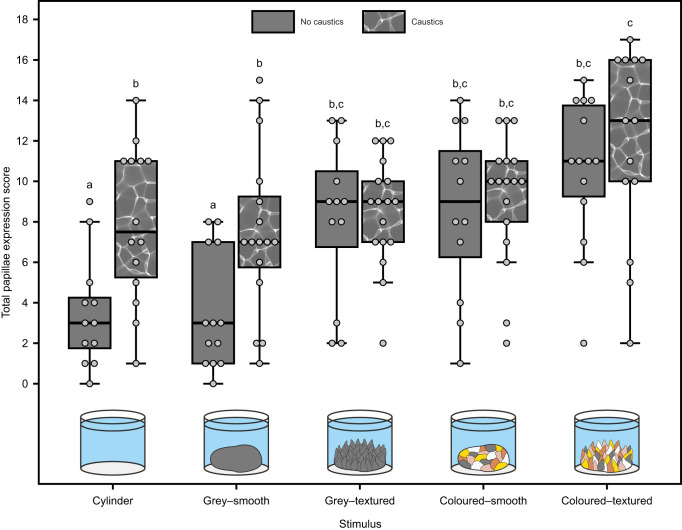
**Papillae expression in cuttlefish (*n*=20).** Total papillae expression score in response to different rock stimuli and exposure to either caustic lighting patterns or static (non-caustic) uniform grey lighting. The total papillae expression score was calculated by adding up the individual papillae expression scores (on a scale from 0 to 3) for six different papilla types (see Fig. 1G for visualisation), thereby ranging from 0 (no papillae expressed) to a maximum score of 18 (all six papilla types fully expressed). Papillae expression was strongest in cuttlefish that were either exposed to caustic lighting or resting next to coloured or textured rocks. Boxplots represent median (horizontal line) and quartiles (upper and lower boundaries) of the data. The whiskers extend to the most extreme measurement within 1.5 times the interquartile range. The jittered grey points represent the raw data, with each point depicting the papillae expression score for an individual cuttlefish in a given combination of rock stimulus and lighting condition. Letters represent statistically similar groups based on Tukey's *post hoc* analysis and *P*<0.05 (Table S2A).

**Fig. 4.**
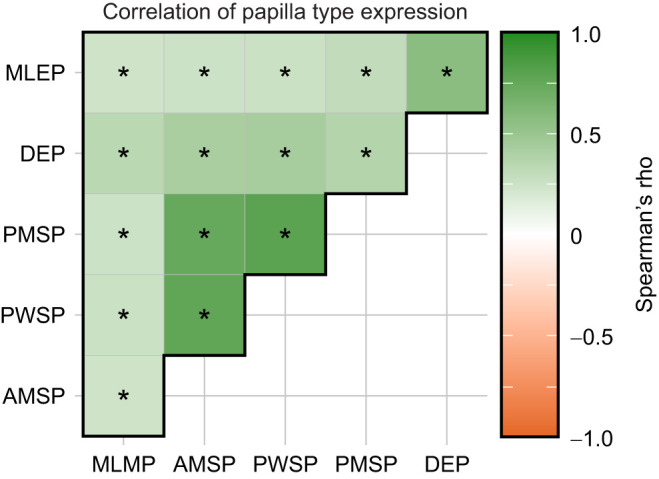
**Correlation coefficients for the expression of the six different papilla types analysed.** Asterisks (*) show significant correlations (*P*<0.05; corrected for multiple testing) between two papilla types. Papilla types: major lateral eye papillae (MLEP); dorsal eye papillae (DEP); posterior mantle spot papillae (PMSP); posterior white square papillae (PWSP); anterior mantle spot papillae (AMSP); and major lateral mantle papillae (MLMP). See Fig. 1G for visualisation of papilla types.

Cuttlefish papillae expression varied based on the type of rock stimulus and the presence or absence of caustics (interaction term between rock stimuli and caustic level: LMM: χ^2^_4_=10.75, *P=*0.03; Fig. 3; Fig. S4). While the absence of a rock (empty cylinder) or the grey–smooth rock elicited the lowest papillae expression (based on *post hoc* analysis; Table S1A), cuttlefish expressed their papillae more strongly either when resting next to coloured or textured rock stimuli or when being exposed to caustic lighting (Fig. 3). For rocks that were either coloured, textured or both, the addition of caustics to the visual scene did not elicit stronger papillae expression in cuttlefish (Fig. 3; Table S1A). Overall, the expression of the six different papilla types analysed was consistently positively correlated, indicating that cuttlefish did not express the different papilla types independently of each other (Fig. 4; see Fig. S4 for individual expression of the different papilla types scored).

**Fig. 5.**
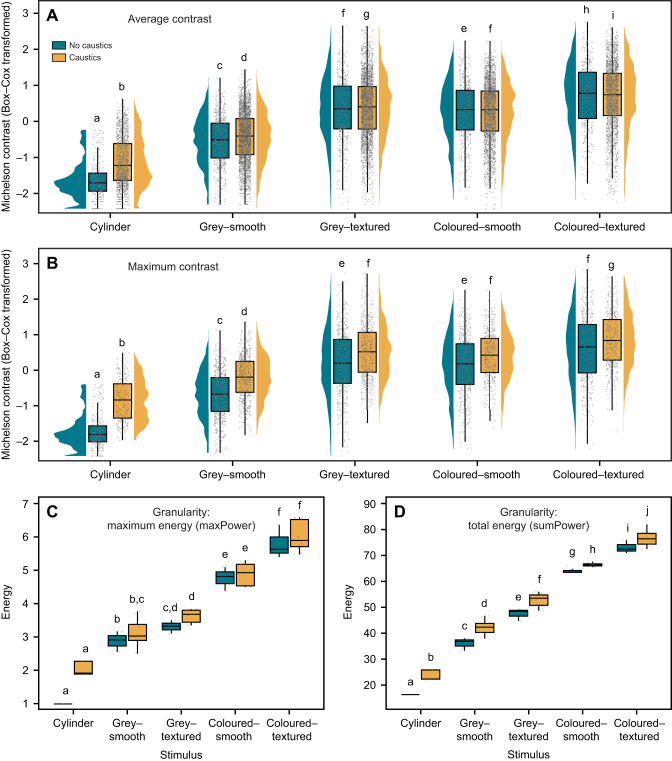
**Contrast and granularity levels of the different rock stimuli in both non-caustic and caustic lighting.** (A) Average contrast. Rock stimuli differed in their average Michelson contrast levels (LMM: *F*_4_=2051.81, *P<*0.001), with caustic exposure further increasing the average contrast of these stimuli (LMM: *F*_1_=26.78, *P<*0.001). (B) Maximum contrast. Rock stimuli differed in their maximum Michelson contrast levels (LMM: *F*_4_=846.01, *P<*0.001), with caustic exposure further increasing the maximum contrast of these stimuli (LMM: *F*_1_=359.91, *P<*0.001). (C) Maximum energy. Rock stimuli differed in their maximum pattern energy (LMM: *F*_4_=281.89, *P<*0.001), with exposure to caustic lighting further altering the rock stimuli's maximum energy (LMM: *F*_1_=8.06, *P=*0.006). (D) Total energy. Rock stimuli differed in their total pattern energy (LMM: *F*_4_=946.37, *P<*0.001), with exposure to caustic lighting further altering the rock stimuli's maximum energy (LMM: *F*_1_=44.12, *P<*0.001). (C,D) See Fig. S3 for visualisation of granularity spectra. (A–D) Boxplots represent median (horizontal line) and quartiles (upper and lower boundaries) of the data, with A and B further showing the density distribution of the data variability. The whiskers extend to the most extreme measurement within 1.5 times the interquartile range. The jittered grey points represent the raw data, with each point representing (A,B) the contrast between two adjacent segments across the rock stimuli surface, (C) the maximum energy measured in a frequency band measured in one of the 78 monochromatic images, or (D) the total pattern energy measured in one of the 78 monochromatic images (see Materials and Methods for detailed description). Letters represent significant groups based on Tukey's *post hoc* analysis and *P*<0.05 (Table S1). (A,C,D) Measurements for rock stimuli under caustic lighting yielded five times as many data points owing to the analysis of five images (as opposed to only one image for stimuli in static, non-caustic lighting).

The rock stimuli varied in both their average (LM: *F*_4_=2051.84, *P<*0.001; Fig. 5A; Table S1B) and maximum contrast levels (LM: *F*_4_=846.01, *P<*0.001; Fig. 5B; Table S1C), with only grey–textured and coloured–smooth rocks sharing similar maximum contrast levels (Fig. 5B; Table S1C). Exposure to caustics further raised both the average (LM: *F*_1_=26.78, *P<*0.001; Fig. 5A; Table S1B) and maximum contrast levels of each rock stimulus (LM: *F*_1_=359.91, *P<*0.001; Fig. 5B; Table S1C). In terms of their granularity (‘spatial information’), the rock stimuli varied in their maximum frequency (LM: *F*_4_=627.71, *P<*0.001), with the grey–smooth rocks having a significantly higher maximum frequency than all other rock stimuli (Fig. S3; Table S1D). In addition, the rock stimuli further varied in both their maximum energy (LM: *F*_4_=281.89, *P<*0.001; Fig. 5C) and total energy (LM: *F*_4_=946.37, *P<*0.001; Fig. 5D), with none of the rock stimuli sharing similar maximum or total energies (Fig. 5C,D; Table S1E,F). Although exposure to caustic lighting did not alter the maximum frequency of any of the rock stimuli (LM: *F*_1_=0.04, *P=*0.838; Fig. S3, Table S1D), it raised both the maximum (LM: *F*_1_=8.06, *P=*0.006; Table S1E) and total energy (LM: *F*_1_=44.12, *P<*0.001; Table S1F) of each rock stimulus (Fig. 5C,D). To determine which of these contrast or granularity metrics best predicted the level of cuttlefish papillae expression, we performed an AIC model selection between five models predicting the total papillae expression score, varying in their explanatory variables (average MC, maximum MC, maximum frequency, maximum energy or total energy), as well as a null model lacking any explanatory variables. The model with the maximum MC as the sole explanatory variable best-predicted cuttlefish papillae expression (Table 1).

**Table 1.** Output of the AIC model selection to determine which contrast or granularity metric best predicted cuttlefish papillae expression

Metric	AICc	ΔAICc	AICcWt	*K*
Maximum Michelson contrast	751.37	0.00	0.91	4
Average Michelson contrast	756.22	4.85	0.08	4
Maximum energy	762.35	10.98	0.00	4
Total energy	768.09	16.72	0.00	4
Maximum frequency	799.78	48.41	0.00	4
Null model	803.96	52.59	0.00	3

AICc, corrected Akaike’s information criterion. ΔAICc, difference in AICc score between the best model and the model compared, with ΔAICc scores of >2 indicating a strong support for best model over the compared one. AICcWt, AICc weight, highlighting the proportion of the total amount of the predictive power provided by the full set of models contained in the model being assessed. *K*, number of parameters in the model.

**Fig. 6.**
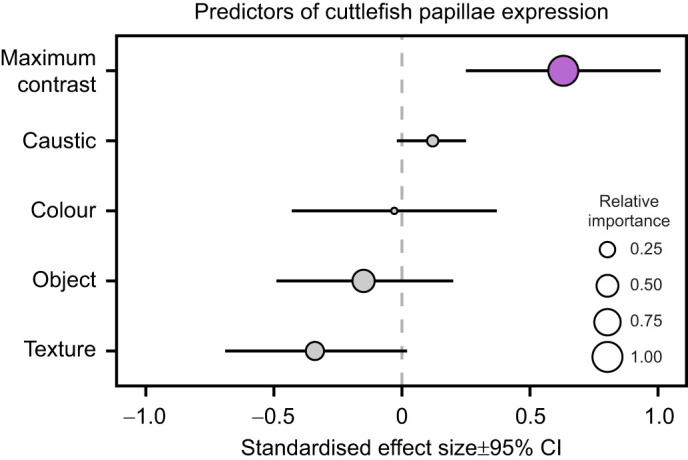
**Predictive visual features used by cuttlefish to inform their three-dimensional camouflage.** Model averaged coefficient estimates from candidate linear mixed models to assess the predictors of cuttlefish papillae expression. Centres of points and error bars represent the model averaged effect sizes and 95% confidence intervals (CI) of each predictor, respectively. Point size denotes the relative importance (RI) of each predictor, with RI values ranging from 0 (predictor appears in the least supported models) to 1 (predictor appears in the best supported models). Predictors that significantly (*P<*0.05) affect the cuttlefish papillae expression are coloured purple.

We then used a model-averaging approach to assess whether any of the visual features that we manipulated across our treatments (presence or absence of rock, surface texture; colouration; presence or absence of caustics) or the best-supported contrast/granularity metric (maximum MC; Table 1) better predicted cuttlefish papillae expression. Maximum MC (*P*=0.001; RI=1.00) was the only significant and best supported predictor of cuttlefish papillae expression (Fig. 6; Table S2), whereas the presence or absence of a rock (*P*=0.404; RI=0.52), its surface texture (*P*=0.064; RI=0.34) and colouration (*P*=0.873; RI=0.12), as well as the presence or absence of caustic lighting (*P*=0.090; RI=0.16), were less-supported predictors of papillae expression in cuttlefish (Fig. 6; Table S2). Overall, cuttlefish papillae expression was thus best predicted by the maximum MC levels in the visual scene.

## DISCUSSION

Cuttlefish erect their papillae in response to the maximum contrast levels in the visual scene. Although we established positive correlations between the degree of papillae expression and various visual features experimentally manipulated throughout this study (e.g. object presence, surface texture and colouration, caustic exposure), as well as different contrast and granularity measurements, papillae expression was best predicted by the maximum MC values within the visual scene of the cuttlefish. Although cuttlefish were thought to express their papillae in response to 3D surface textures (Panetta et al., 2017), our findings suggest that this camouflage response is better predicted by the contrast cues of the visual scene. However, these contrast cues can derive from different object properties, such as intensity differences in its surface colouration or the 3D structure of the surface (where protruding elements create shaded areas that stand in contrast to other elements exposed to direct lighting), as well as dynamic caustic lighting, with the latter being able to increase papillae expression irrespective of the presence or appearance of an object in their visual field. Our findings thus highlight that the maximum contrast levels of the visual scene appear to be the main visual feature eliciting papillae expression in cuttlefish, but these contrast cues can derive from different object properties (e.g. surface patterning or texture) as well as dynamic lighting in the visual scene of the cuttlefish.

Our finding that cuttlefish appear to use contrast cues in their visual scene to inform their papillae expression is consistent with previous research on body pattern expression in cuttlefish (summarised in Chiao et al., 2015). In combination with other visual features, such as spatial scale and edge definition (e.g. Chiao et al., 2013; Kelman et al., 2007), cuttlefish adapt their body patterning as a function of contrast levels in their visual scene (Barbosa et al., 2008; Chiao et al., 2007; Chiao and Hanlon, 2001; Drerup et al., 2024a). Here, the expression and degree of body pattern expression in cuttlefish is thought to be dictated by the highest contrast levels detectable in the visual scene (Chiao et al., 2015), with both background (Barbosa et al., 2008; Buresch et al., 2011; Chiao et al., 2007; Chiao and Hanlon, 2001; Drerup et al., 2024a) and object features (Buresch et al., 2011), as well as dynamic lighting (Drerup et al., 2024a), contributing to these levels. Similarly, cuttlefish in the present study also erected their papillae in response to the maximum contrast cues provided by both physical objects as well as dynamic lighting, highlighting that both body pattern expression and papillae erection are modulated by similar visual information. This correlation raises the question of whether both camouflage responses concur in their expression and underlie the same visual processing mechanisms and neural pathways. Considering the recent advances in the understanding of neural and physiological aspects of cephalopod skin pattern and papillae expression (Gonzalez-Bellido et al., 2018; Montague, 2023; Reiter et al., 2018; Reiter and Laurent, 2020), future research should address the correlation between body patterns and papillae expression in response to visual stimuli from both a behavioural and neural perspective.

As well as establishing that the maximum contrast levels in the visual scene is an important visual feature that cuttlefish use to inform their papillae expression, we also established positive correlations between papillae expression and various spatial frequency measurements (‘granularity’), such as the dominant spatial frequency, the energy within this dominant spatial frequency and the total energy across all spatial frequencies. This is predictable, as adding contrast through chromatic patterns or surface texture will correspondingly also add spatial information to the scene. Although we aimed to minimise the impact and breadth of spatial scales on papillae expression by using roughly equally sized shell fragments and coloured patches across all treatments, spatial scale information has indeed been shown to play an important role in the body pattern expression of cuttlefish (Chiao et al., 2009). It is therefore conceivable that variations in spatial frequencies alone may also affect papillae expression in cuttlefish, and future studies varying the spatial frequencies of the visual scene (while keeping contrast levels consistent) are needed to confirm this hypothesis.

In addition to the spatial scale, cuttlefish also use the spatial orientation of visual cues to inform their camouflage strategies, for example to control their arm postures (Barbosa et al., 2011). As *S. officinalis* possesses nine sets of papillae (Allen et al., 2009) the size and shape of which can be individually altered by a neurally controlled muscular hydrostat system (Allen et al., 2013, 2014; Gonzalez-Bellido et al., 2018), cuttlefish may vary the finer appearance (e.g. the shape or angle) of their individual papillae based on the spatial arrangement of 3D features (or contrast cues deriving from those features) to match their surroundings more closely. In our study, cuttlefish were exposed to evenly distributed and similar sized but randomly arranged 3D textures and chromatic patterns across the entire visual rock stimuli, which resulted in a uniform expression in papillae size expression across all cuttlefish. Therefore, our study did not systematically test whether certain spatial arrangements of surface texture or chromatic patterns affect papillae expression in general, or the finer appearance of papillae in particular. Hence, future research should address whether the spatial size or arrangement of visual features, such as the shape, angle or polarised orientation of 3D surface texture elements (and their corresponding contrast levels), might result in differently shaped or angled papillae. Another research avenue would be to investigate whether local differences in the degree of surface patterning or 3D texture (as well as local differences in the contrast levels deriving from these features) across the visual scene elicit variations in the size, shape and angle of individual papillae across a cuttlefish's body. This approach could inform whether cuttlefish assess various local parts of the visual scene and match the appearance of corresponding body parts to these local conditions, or alternatively use a more global approach by adapting their overall appearance to averaged visual cues across their visual field.

Our study presents a new approach to investigate papillae expression in cephalopods using IR cameras which do not capture the chromatic body patterns of cuttlefish, thereby facilitating visual papillae assessment. By mounting an IR camera above the arena, we were able to analyse a set of six different papilla types from a top-down perspective. However, *S. officinalis* possesses a total of nine papilla types, including hundreds of small dorsal papillae covering the entire dorsal surface (Allen et al., 2009), and our approach did not allow us to analyse all of these papilla types. Future studies should therefore aim to capture images from various angles to better describe the degree to which the nine independently controllable papilla types are erected, and whether their expression varies based on different visual cues. This approach could further be refined using stereo-calibrated cameras paired with photogrammetry techniques (James and Robson, 2012; Karami et al., 2022; Muñoz-Muñoz et al., 2016; Wu et al., 2004), allowing 3D reconstruction of the cuttlefish skin topography, which may also offer the possibility to analyse papillae expression using quantitative rather than qualitative measurements.

Cuttlefish are thought to express their papillae when resting next to textured objects, thereby masquerading as those objects (Panetta et al., 2017) or enhancing cryptic body patterns. In our study, however, exposure to dynamic lighting alone was sufficient to promote papillae expression in cuttlefish, highlighting that this camouflage response can also be adapted in the absence of any textural features in their scene. Assuming that cuttlefish cannot distinguish between contrast cues generated from either static background components or dynamic lighting (Drerup et al., 2024a), papillae expression induced by dynamic lighting might thus result from a perceptual constraint in cuttlefish, suggesting the possibility of dynamic lighting disrupting cuttlefish camouflage. Alternatively, cuttlefish may adopt skin papillae under dynamic lighting to enhance their camouflage. Dynamic lighting increases the complexity of the visual scene, for example by elevating existing contrast levels (Drerup et al., 2024a), adding more visual edges and richer spatial frequencies, and creating false motion cues (Matchette et al., 2018), all of which can disrupt the visual processing abilities of animals (Attwell et al., 2021; Drerup et al., 2025; Matchette et al., 2020; Venables et al., 2022; but see Drerup et al., 2023, 2024b). As obscuring a body's outline is an effective means against many visually guided predators (Osorio and Srinivasan, 1991), cuttlefish may also erect their papillae in dynamic lighting as an additional method of breaking up their body outline and 3D body shape (King et al., 2023; Stevens et al., 2009), thereby further reducing their likelihood of detection. Although our experimental setup exposed cuttlefish to a particular, somewhat artificial combination of water depth, brightness level and caustic patterns, and wild cuttlefish might encounter visual scenes with different contrast levels in their natural habitats, our findings still revealed the extent to which cuttlefish are capable of adopting their visual appearance to a dynamically changing environment. With this in mind, if three-dimensionally textured skin indeed improves camouflage in dynamically lit habitats by breaking up an animal's outline, we should expect animals to be more likely to have textured skin in environments prone to dynamic lighting. Shallow marine reefs, for example, are often exposed to dynamic lighting in the form of caustics, and many cryptic fish species occupying these reefs, such as frog fish of the family Antennariidae or scorpion fish of the family Scorpaenidae, have indeed evolved skin bumps and flaps along the body. In line with our previous findings that cuttlefish adopt disruptive camouflage under dynamic lighting (Drerup et al., 2024a), we propose a systematic review of the correlation between dynamic lighting and morphological features in animals to establish how dynamically lit environments have shaped animal camouflage strategies throughout evolutionary time.

In conclusion, our study demonstrates that cuttlefish papillae expression is largely driven by the maximum contrast cues available in their visual scene, but those contrast cues can derive from different object features as well as dynamic lighting. Although cuttlefish erected their papillae when adjacent to textured rocks, thereby presumably masquerading the appearance of these rocks (Panetta et al., 2017), cuttlefish also expressed their papillae in the absence of any object but when exposed to dynamic lighting. As dynamic lighting increases the visual complexity of the scene, cuttlefish might therefore decrease their likelihood of being detected in dynamically lit environments by erecting their skin papillae to break up their body outline and 3D body shape, highlighting how these cephalopods dynamically adapt their camouflage strategies based on the prevailing visual scene.

## Supplementary Material

10.1242/jexbio.249713_sup1 Supplementary information

Dataset 1. This data file consists of four sheets and contains all data used in the manuscript.

## References

[JEB249713C1] Allen, J. J., Mäthger, L. M., Barbosa, A. and Hanlon, R. T. (2009). Cuttlefish use visual cues to control three-dimensional skin papillae for camouflage. *J. Comp. Physiol. A* 195, 547-555. 10.1007/s00359-009-0430-y 19294390

[JEB249713C2] Allen, J. J., Mäthger, L. M., Buresch, K. C., Fetchko, T., Gardner, M. and Hanlon, R. T. (2010). Night vision by cuttlefish enables changeable camouflage. *J. Exp. Biol.* 213, 3953-3960. 10.1242/jeb.044750 21075936

[JEB249713C3] Allen, J. J., Bell, G. R. R., Kuzirian, A. M. and Hanlon, R. T. (2013). Cuttlefish skin papilla morphology suggests a muscular hydrostatic function for rapid changeability. *J. Morphol.* 274, 645-656. 10.1002/jmor.20121 23378271

[JEB249713C4] Allen, J. J., Bell, G. R. R., Kuzirian, A. M., Velankar, S. S. and Hanlon, R. T. (2014). Comparative morphology of changeable skin papillae in octopus and cuttlefish. *J. Morphol.* 275, 371-390. 10.1002/jmor.20221 24741712

[JEB249713C5] Allen, J. J., Akkaynak, D., Sugden, A. U. and Hanlon, R. T. (2015). Adaptive body patterning, three-dimensional skin morphology and camouflage measures of the slender filefish *Monacanthus tuckeri* on a Caribbean coral reef. *Biol. J. Linn. Soc.* 116, 377-396. 10.1111/bij.12598 PMC8932952 35310331

[JEB249713C6] Allen, M., Poggiali, D., Whitaker, K., Marshall, T. R., van Langen, J. and Kievit, R. A. (2021). Raincloud plots: a multi-platform tool for robust data visualization. *Wellcome Open Res.* 4, 63. 10.12688/wellcomeopenres.15191.2 31069261 PMC6480976

[JEB249713C7] Andrews, P. L. R., Darmaillacq, A.-S., Dennison, N., Gleadall, I. G., Hawkins, P., Messenger, J. B., Osorio, D., Smith, V. J. and Smith, J. A. (2013). The identification and management of pain, suffering and distress in cephalopods, including anaesthesia, analgesia and humane killing. *J. Exp. Mar. Biol. Ecol.* 447, 46-64. 10.1016/j.jembe.2013.02.010

[JEB249713C8] Attwell, J. R., Ioannou, C. C., Reid, C. R. and Herbert-Read, J. E. (2021). Fish avoid visually noisy environments where prey targeting is reduced. *Am. Nat.* 198, 421-432. 10.1086/715434 34403312

[JEB249713C9] Barbosa, A., Mäthger, L. M., Buresch, K. C., Kelly, J., Chubb, C., Chiao, C.-C. and Hanlon, R. T. (2008). Cuttlefish camouflage: the effects of substrate contrast and size in evoking uniform, mottle or disruptive body patterns. *Vis. Res.* 48, 1242-1253. 10.1016/j.visres.2008.02.011 18395241

[JEB249713C10] Barbosa, A., Allen, J. J., Mäthger, L. M. and Hanlon, R. T. (2011). Cuttlefish use visual cues to determine arm postures for camouflage. *Proc. R. Soc. B* 279, 84-90. 10.1098/rspb.2011.0196 PMC3223637 21561967

[JEB249713C11] Bates, D., Mächler, M., Bolker, B. and Walker, S. (2015). Fitting linear mixed-effects models using lme4. *J. Stat. Softw.* 67, 1-48. 10.18637/jss.v067.i01

[JEB249713C12] Bloor, I. S. M., Wearmouth, V. J., Cotterell, S. P., McHugh, M. J., Humphries, N. E., Jackson, E. L., Attrill, M. J. and Sims, D. W. (2013a). Movements and behaviour of European common cuttlefish *Sepia officinalis* in English Channel inshore waters: first results from acoustic telemetry. *J. Exp. Mar. Biol. Ecol.* 448, 19-27. 10.1016/j.jembe.2013.06.013

[JEB249713C13] Bloor, I. S. M., Attrill, M. J. and Jackson, E. L. (2013b). A review of the factors influencing spawning, early life stage survival and recruitment variability in the common cuttlefish (*Sepia officinalis*). *Adv. Mar. Biol.* 65, 1-65. 10.1016/B978-0-12-410498-3.00001-X 23763891

[JEB249713C14] Brauckhoff, M., Wahlberg, M., Haga, J. Å. R., Karlsen, H. E. and Wilson, M. (2020). Embracing their prey at that dark hour: common cuttlefish (*Sepia officinalis*) can hunt in nighttime light conditions. *Front. Physiol.* 11, 525. 10.3389/fphys.2020.00525 32587521 PMC7298144

[JEB249713C15] Brown, P. K. and Brown, P. S. (1958). Visual pigments of the octopus and cuttlefish. *Nature* 182, 1288-1290. 10.1038/1821288a0 13600294

[JEB249713C16] Buresch, K. C., Mäthger, L. M., Allen, J. J., Bennice, C., Smith, N., Schram, J., Chiao, C.-C., Chubb, C. and Hanlon, R. T. (2011). The use of background matching vs. masquerade for camouflage in cuttlefish *Sepia officinalis*. *Vis. Res.* 51, 2362-2368. 10.1016/j.visres.2011.09.009 21964504

[JEB249713C17] Burnham, K. P. and Anderson, D. R. (2002). *Model Selection and Multimodel Inference: A Practical Information-Theoretic Approach*. New York: Springer New York.

[JEB249713C18] Chiao, C.-C. and Hanlon, R. T. (2001). Cuttlefish camouflage: visual perception of size, contrast and number of white squares on artificial checkerboard substrata initiates disruptive coloration. *J. Exp. Biol.* 204, 2119-2125. 10.1242/jeb.204.12.2119 11441053

[JEB249713C19] Chiao, C.-C., Chubb, C. and Hanlon, R. T. (2007). Interactive effects of size, contrast, intensity and configuration of background objects in evoking disruptive camouflage in cuttlefish. *Vis. Res.* 47, 2223-2235. 10.1016/j.visres.2007.05.001 17592739

[JEB249713C20] Chiao, C.-C., Chubb, C., Buresch, K., Siemann, L. and Hanlon, R. T. (2009). The scaling effects of substrate texture on camouflage patterning in cuttlefish. *Vis. Res.* 49, 1647-1656. 10.1016/j.visres.2009.04.002 19362570

[JEB249713C21] Chiao, C.-C., Ulmer, K. M., Siemann, L. A., Buresch, K. C., Chubb, C. and Hanlon, R. T. (2013). How visual edge features influence cuttlefish camouflage patterning. *Vis. Res.* 83, 40-47. 10.1016/j.visres.2013.03.001 23499977

[JEB249713C22] Chiao, C.-C., Chubb, C. and Hanlon, R. T. (2015). A review of visual perception mechanisms that regulate rapid adaptive camouflage in cuttlefish. *J. Comp. Physiol. A* 201, 933-945. 10.1007/s00359-015-0988-5 25701389

[JEB249713C23] Cott, H. B. (1940). *Adaptive Coloration in Animals*. London, UK: Methuen.

[JEB249713C24] Dormann, C. F., Calabrese, J. M., Guillera-Arroita, G., Matechou, E., Bahn, V., Bartoń, K., Beale, C. M., Ciuti, S., Elith, J., Gerstner, K. et al. (2018). Model averaging in ecology: a review of Bayesian, information-theoretic, and tactical approaches for predictive inference. *Ecol. Monogr.* 88, 485-504. 10.1002/ecm.1309

[JEB249713C25] Drerup, C. and Cooke, G. M. (2021). Shoaling behaviour in the European cuttlefish *Sepia officinalis*. *Ethology* 127, 1101-1108. 10.1111/eth.13226

[JEB249713C26] Drerup, C., How, M. J. and Herbert-Read, J. E. (2023). Visual noise from caustic flicker does not affect the hunting success of cuttlefish. *Anim. Behav.* 202, 59-72. 10.1016/j.anbehav.2023.06.002

[JEB249713C27] Drerup, C., Dunkley, K., How, M. J. and Herbert-Read, J. E. (2024a). Cuttlefish adopt disruptive camouflage under dynamic lighting. *Curr. Biol.* 34, 3258-3264. 10.1016/j.cub.2024.06.015 38959882

[JEB249713C28] Drerup, C., How, M. J. and Herbert-Read, J. E. (2024b). Dynamic visual noise has limited influence on the habitat selection and behavioural activity of crustaceans and cephalopods. *Ethology* 130, e13432. 10.1111/eth.13432

[JEB249713C29] Drerup, C., Herbert-Read, J. E. and How, M. J. (2025). Motion after-effects induced by dynamic illumination in crab vision. *Ecol. Evol.* 15, e71426. 10.1002/ece3.71426 40352625 PMC12065077

[JEB249713C30] Fiorito, G., Affuso, A., Basil, J., Cole, A., de Girolamo, P., D'Angelo, L., Dickel, L., Gestal, C., Grasso, F., Kuba, M. et al. (2015). Guidelines for the care and welfare of cephalopods in research – a consensus based on an initiative by CephRes, FELASA and the Boyd Group. *Lab. Anim.* 49, 1-90. 10.1177/0023677215580006 26354955

[JEB249713C31] Godfrey, D., Lythgoe, J. N. and Rumball, D. A. (1987). Zebra stripes and tiger stripes: the spatial frequency distribution of the pattern compared to that of the background is significant in display and crypsis. *Biol. J. Linn. Soc.* 32, 427-433. 10.1111/j.1095-8312.1987.tb00442.x

[JEB249713C32] Gonzalez-Bellido, P. T., Scaros, A. T., Hanlon, R. T. and Wardill, T. J. (2018). Neural control of dynamic 3-dimensional skin papillae for cuttlefish camouflage. *iScience* 1, 24-34. 10.1016/j.isci.2018.01.001 30058000 PMC6059360

[JEB249713C33] Guerra, A. (2006). Ecology of *Sepia officinalis*. *Vie Milieu* 56, 97-107.

[JEB249713C34] Hanlon, R. T. and Messenger, J. B. (1988). Adaptive coloration in young cuttlefish (*Sepia officinalis* L.): the morphology and development of body patterns and their relation to behaviour. *Philos. Trans. R. Soc. B* 320, 437-487.

[JEB249713C35] Hothorn, T., Bretz, F. and Westfall, P. (2008). Simultaneous inference in general parametric models. *Biom. J.* 50, 346-363. 10.1002/bimj.200810425 18481363

[JEB249713C36] James, M. R. and Robson, S. (2012). Straightforward reconstruction of 3D surfaces and topography with a camera: accuracy and geoscience application. *J. Geophys. Res.* 117, F03017. 10.1029/2011JF002289

[JEB249713C37] John, L., Santon, M. and Michiels, N. K. (2024). Scorpionfish adjust skin pattern contrast on different backgrounds. *Ecol. Evol.* 14, e11124. 10.1002/ece3.11124 38476704 PMC10928359

[JEB249713C38] Karami, A., Menna, F. and Remondino, F. (2022). Combining photogrammetry and photometric stereo to achieve precise and complete 3D reconstruction. *Sensors* 22, 8172. 10.3390/s22218172 36365869 PMC9654855

[JEB249713C39] Kelman, E. J., Baddeley, R. J., Shohet, A. J. and Osorio, D. (2007). Perception of visual texture and the expression of disruptive camouflage by the cuttlefish, *Sepia officinalis*. *Proc. R. Soc. B* 274, 1369-1375. 10.1098/rspb.2007.0240 PMC2176201 17389219

[JEB249713C40] King, J., Hemmi, J. M. and Kelley, J. L. (2023). Camouflage using three-dimensional surface disruption. *Biol. Lett.* 19, 20220596. 10.1098/rsbl.2022.0596 37528728 PMC10394419

[JEB249713C41] Lock, J. A. and Andrews, J. H. (1992). Optical caustics in natural phenomena. *Am. J. Phys.* 60, 397-407. 10.1119/1.16891

[JEB249713C42] Lüdecke, D., Ben-Shachar, M. S., Patil, I., Waggoner, P. and Makowski, D. (2021). performance: an R package for assessment, comparison and testing of statistical models. *J. Open Source Softw.* 6, 3139. 10.21105/joss.03139

[JEB249713C43] Mark, C. J., O'Hanlon, J. C. and Holwell, G. I. (2022). Camouflage in lichen moths: field predation experiments and avian vision modelling demonstrate the importance of wing pattern elements and background for survival. *J. Anim. Ecol.* 91, 2358-2369. 10.1111/1365-2656.13817 36169598 PMC10092008

[JEB249713C44] Marshall, N. J. and Messenger, J. B. (1996). Colour-blind camouflage. *Nature* 382, 408-409. 10.1038/382408b08684479

[JEB249713C45] Matchette, S. R., Cuthill, I. C. and Scott-Samuel, N. E. (2018). Concealment in a dynamic world: dappled light and caustics mask movement. *Anim. Behav.* 143, 51-57. 10.1016/j.anbehav.2018.07.003

[JEB249713C46] Matchette, S. R., Cuthill, I., Cheney, K., Marshall, N. and Scott-Samuel, N. (2020). Underwater caustics disrupt prey detection by a reef fish. *Proc. R. Soc. B* 287, 20192453. 10.1098/rspb.2019.2453 PMC7209061 32228405

[JEB249713C47] Mäthger, L. M., Barbosa, A., Miner, S. and Hanlon, R. T. (2006). Color blindness and contrast perception in cuttlefish (*Sepia officinalis*) determined by a visual sensorimotor assay. *Vis. Res.* 46, 1746-1753. 10.1016/j.visres.2005.09.035 16376404

[JEB249713C48] McFarland, W. N. and Loew, E. R. (1983). Wave produced changes in underwater light and their relations to vision. *Environ. Biol. Fish.* 8, 173-184. 10.1007/BF00001083

[JEB249713C49] McHugh, M. L. (2012). Interrater reliability: the kappa statistic. *Biochem. Med.* 22, 276-282. 10.11613/BM.2012.031 PMC3900052 23092060

[JEB249713C50] Merilaita, S. and Stevens, M. (2011). Crypsis through background matching. In *Animal Camouflage: Mechanisms and Function* (ed. M. Stevens and S. Merilaita), pp. 17-33. Cambridge: Cambridge University Press.

[JEB249713C51] Merilaita, S., Scott-Samuel, N. E. and Cuthill, I. C. (2017). How camouflage works. *Philos. Trans. R. Soc. B* 372, 20160341. 10.1098/rstb.2016.0341 PMC5444062 28533458

[JEB249713C52] Michalis, C., Scott-Samuel, N. E., Gibson, D. P. and Cuthill, I. C. (2017). Optimal background matching camouflage. *Proc. R. Soc. B* 284, 20170709. 10.1098/rspb.2017.0709 PMC5524497 28701559

[JEB249713C53] Montague, T. G. (2023). Neural control of cephalopod camouflage. *Curr. Biol.* 33, R1095-R1100. 10.1016/j.cub.2023.08.095 37875091

[JEB249713C54] Muñoz-Muñoz, F., Quinto-Sánchez, M. and González-José, R. (2016). Photogrammetry: a useful tool for three-dimensional morphometric analysis of small mammals. *J. Zool. Syst. Evol. Res.* 54, 318-325. 10.1111/jzs.12137

[JEB249713C55] Osorio, D. and Srinivasan, M. V. (1991). Camouflage by edge enhancement in animal coloration patterns and its implications for visual mechanisms. *Proc. R. Soc. B* 244, 81-85. 10.1098/rspb.1991.0054 1679552

[JEB249713C56] Panetta, D., Buresch, K. and Hanlon, R. T. (2017). Dynamic masquerade with morphing three-dimensional skin in cuttlefish. *Biol. Lett.* 13, 20170070. 10.1098/rsbl.2017.0070 28356412 PMC5377043

[JEB249713C57] Quach, L., Miller, A. E., Hogan, B. G. and Stoddard, M. C. (2021). Egg patterns as identity signals in colonial seabirds: a comparison of four alcid species. *J. Exp. Zool. B Mol. Dev. Evol.* 336, 595-605. 10.1002/jez.b.22945 32400035

[JEB249713C58] Randall, J. E. and Randall, H. A. (1960). Examples of mimicry and protective resemblance in tropical marine fishes. *Bull. Mar. Sci.* 10, 444-480.

[JEB249713C59] Reiter, S. and Laurent, G. (2020). Visual perception and cuttlefish camouflage. *Curr. Opin. Neurobiol.* 60, 47-54. 10.1016/j.conb.2019.10.010 31837480

[JEB249713C60] Reiter, S., Hülsdunk, P., Woo, T., Lauterbach, M. A., Eberle, J. S., Akay, L. A., Longo, A., Meier-Credo, J., Kretschmer, F., Langer, J. D. et al. (2018). Elucidating the control and development of skin patterning in cuttlefish. *Nature* 562, 361-366. 10.1038/s41586-018-0591-3 30333578 PMC6217936

[JEB249713C61] Rohr, V. A., Volkmer, T., Metzler, D. and Küpper, C. (2021). Neoptile feathers contribute to outline concealment of precocial chicks. *Sci. Rep.* 11, 5483. 10.1038/s41598-021-84227-4 33750790 PMC7943783

[JEB249713C62] Schneider, C. A., Rasband, W. S. and Eliceiri, K. W. (2012). NIH Image to ImageJ: 25 years of image analysis. *Nat. Methods* 9, 671. 10.1038/nmeth.2089 22930834 PMC5554542

[JEB249713C63] Stevens, M., Cuthill, I. C., Windsor, A. M. M. and Walker, H. J. (2006). Disruptive contrast in animal camouflage. *Proc. R. Soc. B* 273, 2433-2438. 10.1098/rspb.2006.3614 PMC1634902 16959632

[JEB249713C64] Stevens, M., Winney, I. S., Cantor, A. and Graham, J. (2009). Outline and surface disruption in animal camouflage. *Proc. R. Soc. B* 276, 781-786. 10.1098/rspb.2008.1450 PMC2660950 19019788

[JEB249713C65] Stoddard, M. C. and Osorio, D. (2019). Animal coloration patterns: linking spatial vision to quantitative analysis. *Am. Nat.* 193, 164-186. 10.1086/701300 30720360

[JEB249713C66] Stoddard, M. C. and Stevens, M. (2010). Pattern mimicry of host eggs by the common cuckoo, as seen through a bird's eye. *Proc. R. Soc. B* 277, 1387-1393. 10.1098/rspb.2009.2018 PMC2871939 20053650

[JEB249713C67] Stubbs, A. L. and Stubbs, C. W. (2016). Spectral discrimination in color blind animals via chromatic aberration and pupil shape. *Proc. Nat. Acad. Sci.* 113, 8206-8211. 10.1073/pnas.1524578113 27382180 PMC4961147

[JEB249713C68] Šulc, M., Troscianko, J., Štětková, G., Hughes, A. E., Jelínek, V., Capek, M. and Honza, M. (2019). Mimicry cannot explain rejection type in a host–brood parasite system. *Anim. Behav.* 155, 111-118. 10.1016/j.anbehav.2019.05.021

[JEB249713C69] Troscianko, J. and Stevens, M. (2015). Image calibration and analysis toolbox – a free software suite for objectively measuring reflectance, colour and pattern. *Methods Ecol. Evol.* 6, 1320-1331. 10.1111/2041-210X.12439 27076902 PMC4791150

[JEB249713C70] van den Berg, C. P. (2018). Pattern energy (granularity) analysis. Empirical Imaging. https://www.empiricalimaging.com/knowledge-base/pattern-energy-granularity-analysis/ (accessed 05/03/2025).

[JEB249713C71] Venables, S. V., Drerup, C., Powell, S. B., Marshall, N. J., Herbert-Read, J. E. and How, M. J. (2022). Polarization vision mitigates visual noise from flickering light underwater. *Sci. Adv.* 8, eabq2770. 10.1126/sciadv.abq2770 36083913 PMC9462692

[JEB249713C72] Wickham, H. (2016). *ggplot2: Elegant Graphics for Data Analysis*. New York: Springer.

[JEB249713C73] Wu, J., Tillett, R., McFarlane, N., Ju, X., Siebert, J. P. and Schofield, P. (2004). Extracting the three-dimensional shape of live pigs using stereo photogrammetry. *Comput. Electron. Agric.* 44, 203-222. 10.1016/j.compag.2004.05.003

[JEB249713C74] Wu, J. J.-S., Hung, A., Lin, Y.-C. and Chiao, C.-C. (2020). Visual attack on the moving prey by cuttlefish. *Front. Physiol.* 11, 648. 10.3389/fphys.2020.00648 32625116 PMC7315006

[JEB249713C75] Wuthrich, K. L., Nagel, A. and Swierk, L. (2022). Rapid body color change provides lizards with facultative crypsis in the eyes of their avian predators. *Am. Nat.* 199, 277-290. 10.1086/717678 35077274

[JEB249713C76] Yu, H., Lin, Z. and Xiao, F. (2024). Role of body size and shape in animal camouflage. *Ecol. Evol.* 14, e11434. 10.1002/ece3.11434 38746542 PMC11090776

[JEB249713C77] Zylinski, S., Osorio, D. and Shohet, A. (2009). Cuttlefish camouflage: context-dependent body pattern use during motion. *Proc. R. Soc. B* 276, 3963-3969. 10.1098/rspb.2009.1083 PMC2825777 19692411

