# Supplementary material for: Visual contrast from background features and dynamic illumination contributes to three-dimensional camouflage in cuttlefish
Source: J Exp Biol. 2025 Aug 15;228(16):jeb249713. doi: 10.1242/jeb.249713 (PMC12401539; doi:10.1242/jeb.249713)
Supplement: Supplementary information [file jexbio-228-249713-s1.pdf]

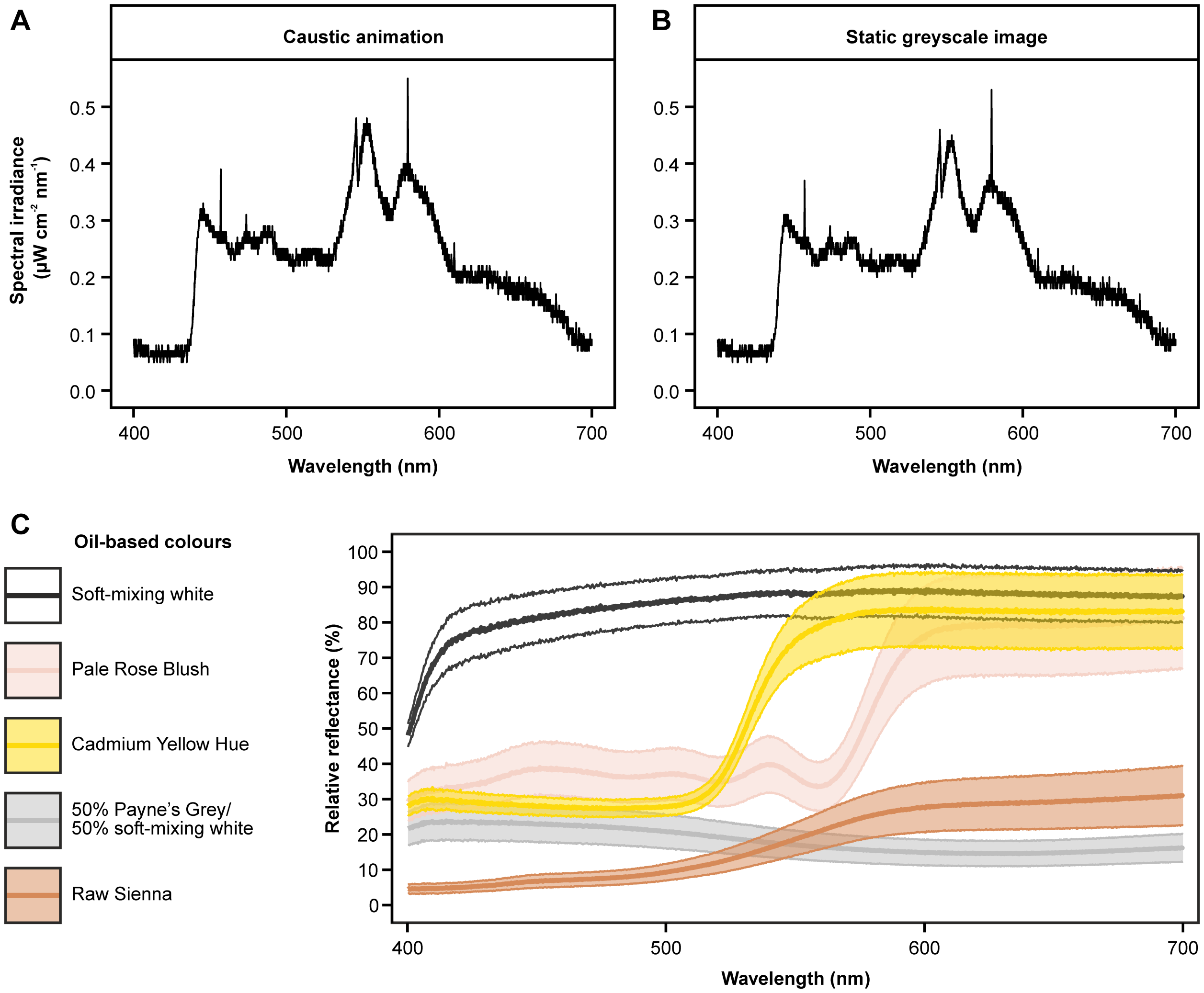

Fig. S1. Extended methods. (A-B) Mean spectral irradiance (μW cm^{-2} nm^{-1}) of (A) the caustic animation and (B) the static greyscale image (uint8 value = 87). Measurements were performed with a spectrometer (USB2000) coupled to a 400 μm bare optic fibre (R400-7-UV-VIS) calibrated using a calibrated light source (DH-2000; all Ocean Insight, Orlando, FL, USA). (C) Oil-based colours used to emulate shades ubiquitously found in shallow marine habitats. Reflectance spectra of each colour were measured for 9 replicates (randomly chosen spots on the rock stimuli) using a spectrometer (OceanHDX) coupled to a 400 μm bare optic fibre (R400-7-UV-VIS) and a halogen light source (HL-2000; all Ocean Insight, Orlando, USA).

Journal of Experimental Biology • Supplementary information

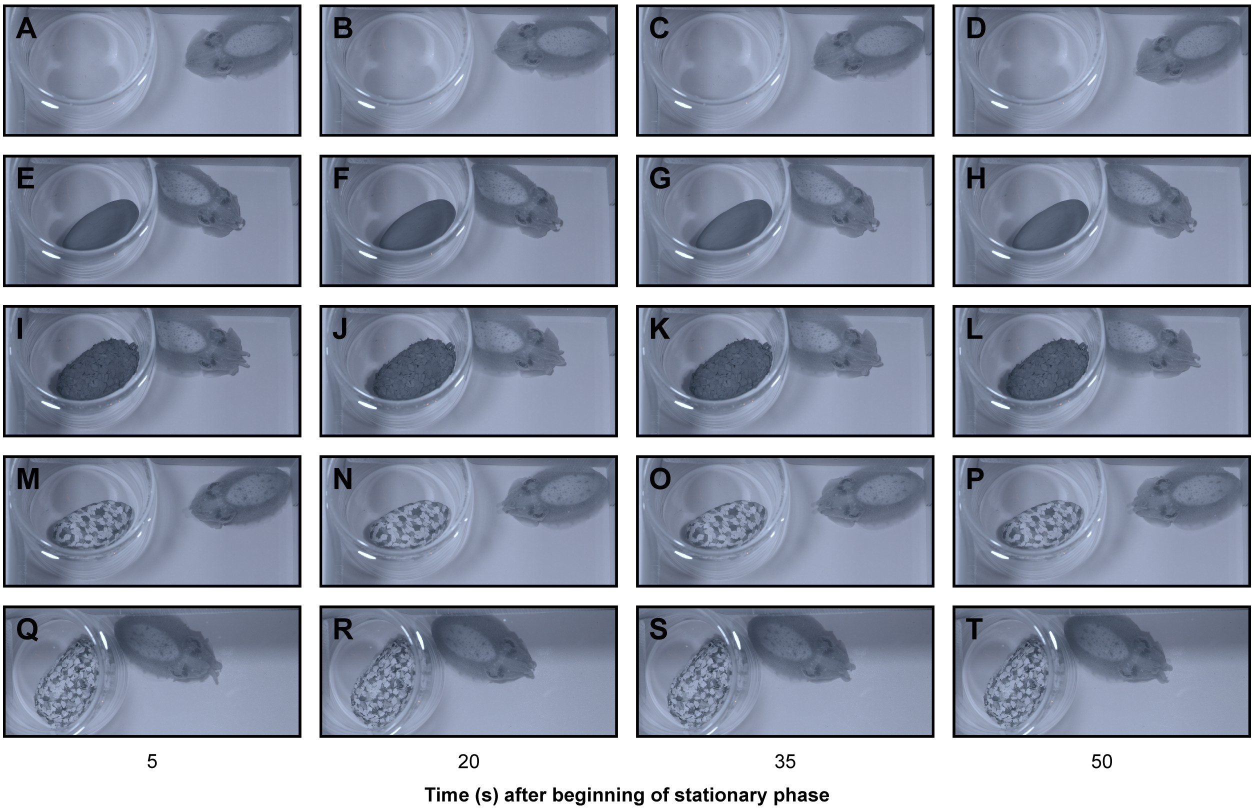

Fig. S2. Stability of cuttlefish papillae expression over time. (A-T) Infrared images of the same cuttlefish resting next to (A-D) the empty glass cylinder, (E-H) a grey-smooth rock, (I-L) a grey-textured rock, (M-P) a coloured-smooth rock, and (Q-T) a coloured-textured rock in caustic lighting, taken 5 s, 20s, 35s, and 50s after the beginning of the stationary phase. Cuttlefish express their papillae immediately after resting next to a stimulus and maintain their papillae expression with little to no variation over time.

Journal of Experimental Biology • Supplementary information

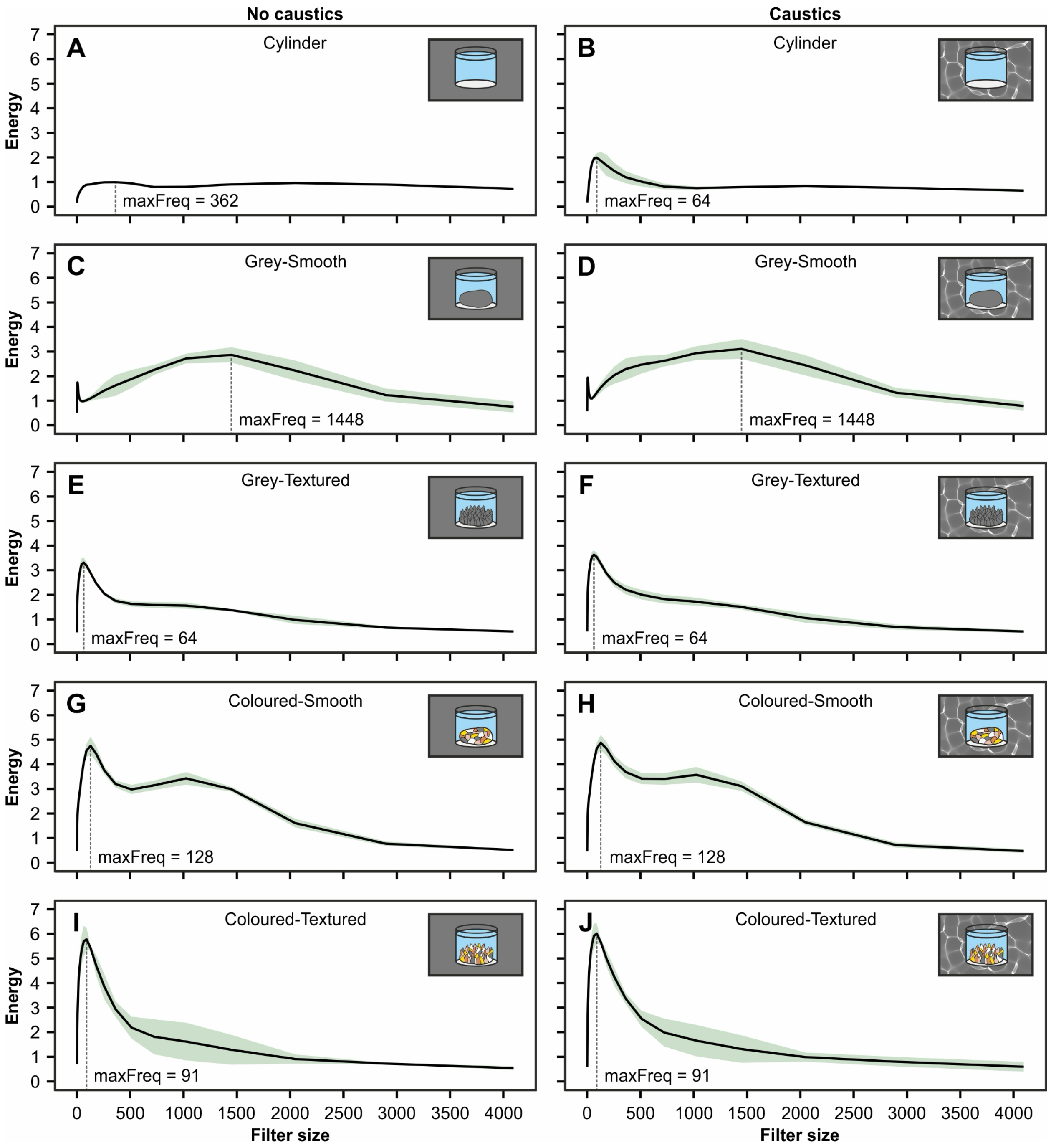

Fig. S3. Granularity spectra of the five rock stimuli in non-caustic or caustic lighting conditions. Measurements were taken for filter sizes ranging from 1 pixel to 4096 pixels with stepwise increments of multiplies of √2. Black lines indicate the average energy per filter size while the green area depicts the standard variation. Dashed grey lines indicate 'maxFreq', the filter size with the highest measured energy.

Journal of Experimental Biology • Supplementary information

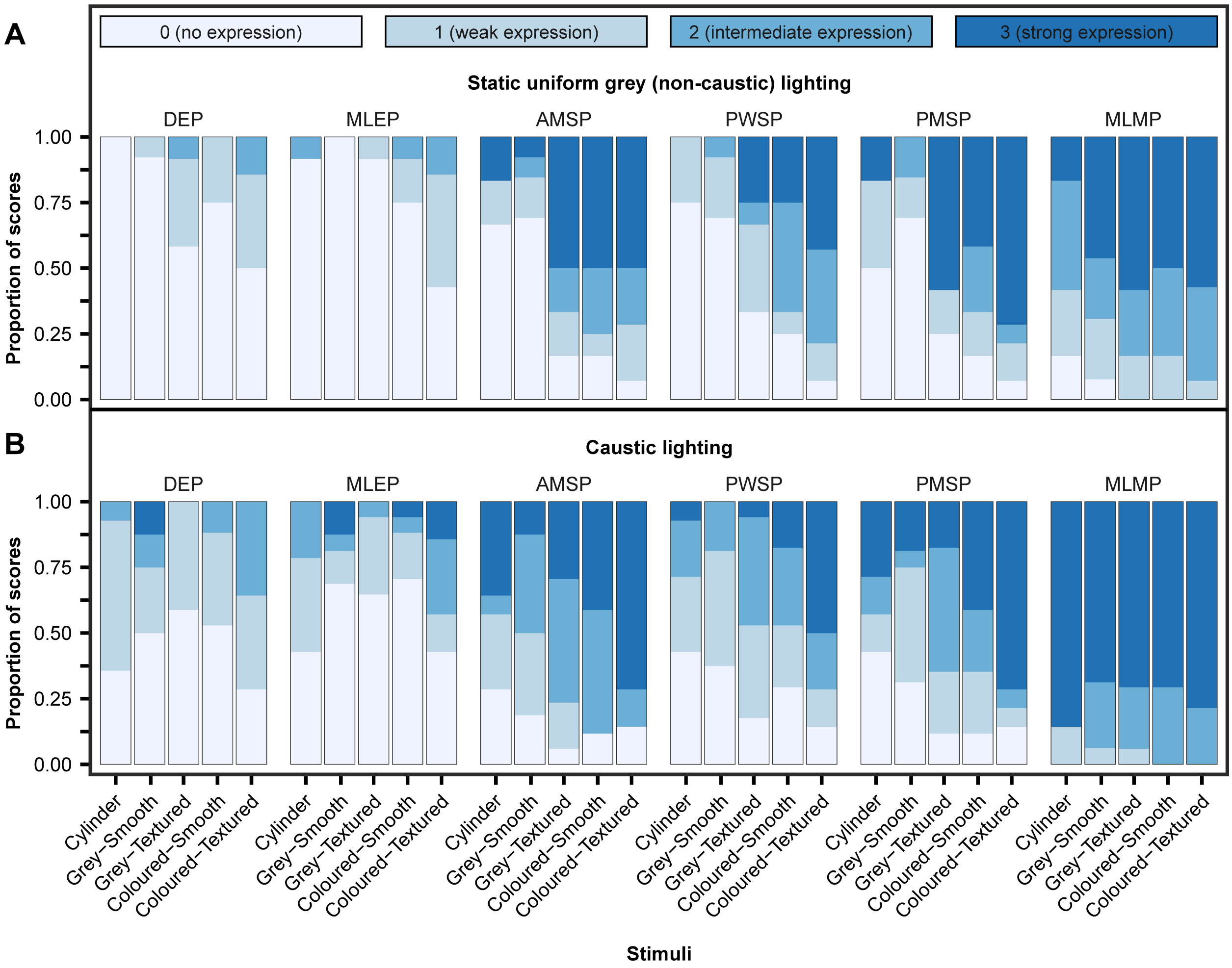

Fig. S4. Individual papillae expression in cuttlefish. Papillae expression score in response to different rock stimuli and exposure to either (A) static (non-caustic) uniform grey lighting or (B) caustic lighting patterns. Six papilla types (DEP [dorsal eye papillae]; MLEP [major lateral eye papillae]; AMSP [anterior mantle spot papillae]; PWSP [posterior white square papillae]; PMSP [posterior mantle spot papillae]; and MLMP [major lateral mantle papillae]; see Fig. 1G for visualisation) were scored using the following scale: 0 (no expression), 1 (weak expression; approximately 1/3 extended), 2 (intermediate expression; approximately 2/3 extended), 3 (strong expression, fully extended), see Fig. 1H-K for visualisation.

Journal of Experimental Biology • Supplementary information

Table S1. Post-hoc analysis of papillae expression experiment as well as the contrast and granularity measurements. Estimated marginal means for the combinations of rock stimuli and caustic conditions from (A) the papillae expression experiment, (B) the average Michelson contrast analysis, (C) the maximum Michelson contrast analysis, (D) the maximum frequency analysis, (E) the maximum energy analysis, and (F) the total energy analysis.

	Rock stimuli	Caustic condition	emmean	SE	df	lower CI	upper CI	subset
**A)**	**Papillae expression experiment**							
	Cylinder	No_caustics	3.69	1.01	103	1.69	5.69	a
	Cylinder	Caustics	7.82	0.95	93	5.94	9.70	b
	Grey-Smooth	No_caustics	3.51	0.98	98	1.57	5.44	a
	Grey-Smooth	Caustics	7.50	0.89	85	5.73	9.28	b
	Grey-Textured	No_caustics	8.24	1.01	102	6.23	10.24	bc
	Grey-Textured	Caustics	8.53	0.87	82	6.79	10.27	bc
	Coloured-Smooth	No_caustics	8.65	1.01	103	6.65	10.66	bc
	Coloured-Smooth	Caustics	9.24	0.87	82	7.50	10.97	bc
	Coloured-Textured	No_caustics	10.61	0.95	93	8.73	12.49	bc
	Coloured-Textured	Caustics	11.75	0.95	93	9.87	13.63	c
**B)**	**Average Michelson contrast analysis**							
	Cylinder	No_caustics	-1.28	0.02	14545	-1.35	-1.22	a
	Cylinder	Caustics	-1.19	0.02	14545	-1.25	-1.14	b
	Grey-Smooth	No_caustics	-0.58	0.02	14545	-0.58	-0.47	c
	Grey-Smooth	Caustics	-0.46	0.01	14545	-0.47	-0.40	d
	Grey-Textured	No_caustics	0.28	0.02	14545	0.23	0.34	f
	Grey-Textured	Caustics	0.37	0.01	14545	0.33	0.41	g
	Coloured-Smooth	No_caustics	0.18	0.02	14545	0.12	0.24	e
	Coloured-Smooth	Caustics	0.27	0.01	14545	0.23	0.31	f
	Coloured-Textured	No_caustics	0.60	0.02	14545	0.54	0.66	h
	Coloured-Textured	Caustics	0.69	0.02	14545	0.65	0.74	i
**C)**	**Maximum Michelson contrast analysis**							
	Cylinder	No_caustics	-1.49	0.03	4858	-1.58	-1.40	a
	Cylinder	Caustics	-1.08	0.03	4858	-1.17	-0.99	b
	Grey-Smooth	No_caustics	-0.65	0.02	4858	-0.72	-0.58	c
	Grey-Smooth	Caustics	-0.24	0.02	4858	-0.31	-0.17	d
	Grey-Textured	No_caustics	0.15	0.02	4858	0.08	0.22	e
	Grey-Textured	Caustics	0.56	0.02	4858	0.49	0.63	f
	Coloured-Smooth	No_caustics	0.05	0.03	4858	-0.02	0.12	e
	Coloured-Smooth	Caustics	0.46	0.03	4858	0.39	0.53	f
	Coloured-Textured	No_caustics	0.51	0.03	4858	0.44	0.59	f
	Coloured-Textured	Caustics	0.92	0.03	4858	0.85	1.00	g
**D)**	**Maximum frequency analysis**							
	Cylinder	No_caustics	139.9	45.1	72	9.75	270	a
	Cylinder	Caustics	134.0	38.6	72	22.60	245	a
	Grey-Smooth	No_caustics	1358.8	32.4	72	1265.06	1452	b
	Grey-Smooth	Caustics	1352.9	22.6	72	1287.62	1418	b
	Grey-Textured	No_caustics	66.8	32.4	72	-26.92	160	a
	Grey-Textured	Caustics	60.9	22.6	72	-4.36	126	a
	Coloured-Smooth	No_caustics	126.6	32.4	72	32.92	220	a
	Coloured-Smooth	Caustics	120.8	22.6	72	55.48	186	a
	Coloured-Textured	No_caustics	83.6	32.4	72	-10.11	177	a
	Coloured-Textured	Caustics	77.8	22.6	72	12.45	143	a

Journal of Experimental Biology • Supplementary information

Table S1. Continued.

	Rock stimuli	Caustic condition	emmean	SE	df	lower CI	upper CI	subset
**E)**	**Maximum energy analysis**							
	Cylinder	No_caustics	1.62	0.16	72	1.16	2.09	a
	Cylinder	Caustics	1.91	0.14	72	1.51	2.31	a
	Grey-Smooth	No_caustics	2.83	0.12	72	2.49	3.17	b
	Grey-Smooth	Caustics	3.12	0.08	72	2.89	3.35	bc
	Grey-Textured	No_caustics	3.34	0.12	72	3.00	3.67	cd
	Grey-Textured	Caustics	3.63	0.08	72	3.39	3.86	d
	Coloured-Smooth	No_caustics	4.62	0.12	72	4.28	4.96	e
	Coloured-Smooth	Caustics	4.91	0.08	72	4.68	5.15	e
	Coloured-Textured	No_caustics	5.74	0.12	72	5.40	6.08	f
	Coloured-Textured	Caustics	6.03	0.08	72	5.80	6.26	f
**F)**	**Total energy analysis**							
	Cylinder	No_caustics	18.6	1.10	72	15.4	21.8	a
	Cylinder	Caustics	23.3	0.94	72	20.5	26.0	b
	Grey-Smooth	No_caustics	37.3	0.79	72	35.0	39.6	c
	Grey-Smooth	Caustics	41.9	0.55	72	40.3	43.5	d
	Grey-Textured	No_caustics	48.1	0.79	72	45.8	50.4	e
	Grey-Textured	Caustics	52.7	0.55	72	51.1	54.3	f
	Coloured-Smooth	No_caustics	62.1	0.79	72	59.8	64.4	g
	Coloured-Smooth	Caustics	66.7	0.55	72	65.1	68.3	h
	Coloured-Textured	No_caustics	72.1	0.79	72	69.8	74.4	i
	Coloured-Textured	Caustics	76.7	0.55	72	75.1	78.3	j

Table S2. **Output of model averaging**. Model averaged coefficient estimates from candidate linear mixed-effects model to establish the predictors of papillae expression in cuttlefish, based on the 95% candidate set of models. All estimates are reported with the lower and upper 95% confidence intervals (CI) as well as their corresponding relative importance values (ranging from 0 to 1). Abbreviations: SE = Standard error; RI = Relative importance.

Parameter	Estimate	95% CI			SE	z	*P*	RI
Maximum Michelson contrast	0.63	0.25	–	1.01	0.19	3.25	0.001	1.00
Object presence	-0.15	-0.49	–	0.20	0.17	0.83	0.404	0.52
Texture	-0.34	-0.69	–	0.02	0.18	1.85	0.064	0.34
Colouration	-0.03	-0.43	–	0.37	0.20	0.16	0.873	0.12
Caustics	0.12	-0.02	–	0.25	0.07	1.70	0.090	0.16

Dataset 1. This data file consists of four sheets and contains all data used in the manuscript.

- **Papillae_expression (Sheet 1):**
  - Trial number: Trial number within experiment
  - Animal_ID: Individual ID used for cuttlefish in this experiment
  - Segment: Each trial included the presentation of 5 different rock stimuli. Segment refers to the order number of a treatment within a trial
  - Rock_ID: ID used for the five different rock stimuli
  - Rock_names: Names of the five different rock stimuli presented
  - Caustic_condition: Cuttlefish were either exposed to caustic lighting ('Caustics') or a static grey image ('No_caustics')
  - Image: Depending on whether or not the cuttlefish rested long in close proximity to a rock stimulus, a segment resulted in either an image (1) used for analysis, or no image (0)
  - DEP: Score for expression of dorsal eye papillae (on a scale from 0 to 3)
  - MLEP: Score for expression of major lateral eye papillae (on a scale from 0 to 3)
  - AMSP: Score for expression of anterior mantle spot papillae (on a scale from 0 to 3)
  - PWSP: Score for expression of posterior white square papillae (on a scale from 0 to 3)
  - PMSP: Score for expression of posterior mantle spot papillae (on a scale from 0 to 3)
  - MLMP: Score for expression of major lateral mantle papillae (on a scale from 0 to 3)
  - Score_total: Total papillae expression score, calculated by adding the six individual papillae scores (can range from 0 to 18)
  - Texture: Texture level of the presented rock stimuli (on a scale from 1 to 3)
  - Colour: Colour level of the presented rock stimuli (on a scale from 1 to 3)
  - Object: Showing whether a rock stimulus was absent (1) or present (2)
  - Caustic: Caustic level, either no caustic lighting (1) or caustic lighting (2)
  - Contrast_average: Mean average contrast value of the presented rock stimuli in the presented caustic lighting condition
  - Contrast_maximum: Mean maximum contrast value of the presented rock stimuli in the presented caustic lighting condition
  - maxPower: Median frequency with the maximum measured pattern energy of the presented rock stimuli in the presented caustic lighting condition
  - maxFreq: Mean maximum energy within the maximum frequency of the presented rock stimuli in the presented caustic lighting condition
  - sumPower: Mean total energy across all frequency bands of the presented rock stimuli in the presented caustic lighting condition

- **Average_contrast (Sheet 2):**
  - Rock: Names of the five different rock stimuli
  - Caustic_condition: Rocks were either exposed to caustic lighting ('Caustic') or a static grey image ('No_caustic')
  - Contrast: Michelson contrast measured between two neighbouring transect segments

- **Maximum_contrast (Sheet 3):**
  - Rock: Names of the five different rock stimuli
  - Caustic_condition: Rocks were either exposed to caustic lighting ('Caustic') or a static grey image ('No_caustic')
  - Contrast: Michelson contrast measured between two neighbouring transect segments

Journal of Experimental Biology • Supplementary information

- **Granularity (Sheet 4):**
  - Rock: Names of the five different rock stimuli
  - Caustic_condition: Rocks were either exposed to caustic lighting ('Caustic') or a static grey image ('No_caustic')
  - maxFreq: Maximum frequency measured for a monochromatic image of a rock stimuli in either caustic or non-caustic lighting
  - maxPower: Energy within the maximum frequency measured for a monochromatic image of a rock stimuli in either caustic or non-caustic lighting
  - sumPower: Total energy measured across all frequency bands measured for a monochromatic image of a rock stimuli in either caustic or non-caustic lighting

Available for download at
https://journals.biologists.com/jeb/article-lookup/doi/10.1242/jeb.249713#supplementary-data

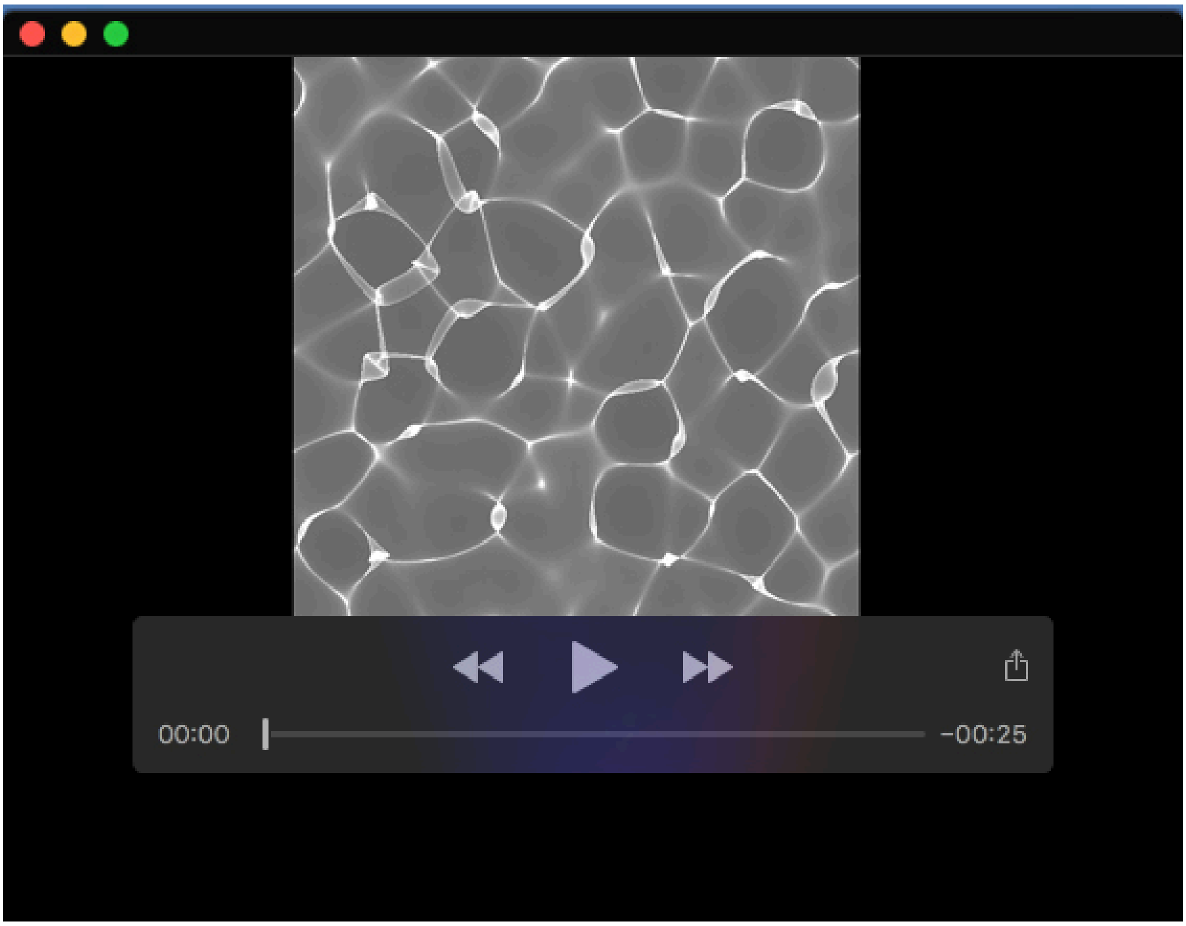

Movie 1. Caustic animation used in this study. The looping animation of a caustic light pattern consisted of 200 unique frames and was rendered using Caustics Generator Pro software (Dual Heights; www.dualheights.se/caustics/).
